# Milestones in the Elucidation of Heme Biosynthesis

**DOI:** 10.1021/acs.chemrev.6c00368

**Published:** 2026-08-24

**Authors:** Harry A. Dailey

**Affiliations:** Department of Microbiology, Department of Biochemistry & Molecular Biology, 1355 University of Georgia, Athens, Georgia 30602, United States

## Abstract

Heme (protoheme IX) is an essential cofactor in biology, serving diverse roles including, but not limited to, one-electron reactions, oxygen transport, catalysis, and signaling. The elucidation of its biosynthetic pathway represents one of the more remarkable scientific accomplishments of the past century. Early 19th-century chemists first isolated, named and characterized porphyrins, while their structural determination by Fischer in the 1920s earned a Nobel Prize. The mid-20th century ushered in isotopic tracer studies which revealed glycine and succinyl-CoA as the starting substrates for the pathway to heme. Identification of porphobilinogen (PBG) as an early pathway intermediate led to the identification of 5-aminolevulinic acid (ALA) as the first committed pathway precursor. Subsequent decades brought the recognition of porphyrinogens as true pathway intermediates, the sequence of decarboxylation and oxidation steps, the identification of the glutamate-based pathway to ALA in plants and most bacteria, the characterization of all pathway enzymes, the discovery of alternate bacterial pathways, and identification of multiprotein complexes. This review traces the historical progression of discoveries, highlighting both the breakthroughs and missteps that shape the current view of heme biosynthesis, and underscores the interplay of chemistry, physiology, and molecular biology in unraveling this ancient metabolic pathway.

## Graphical abstract

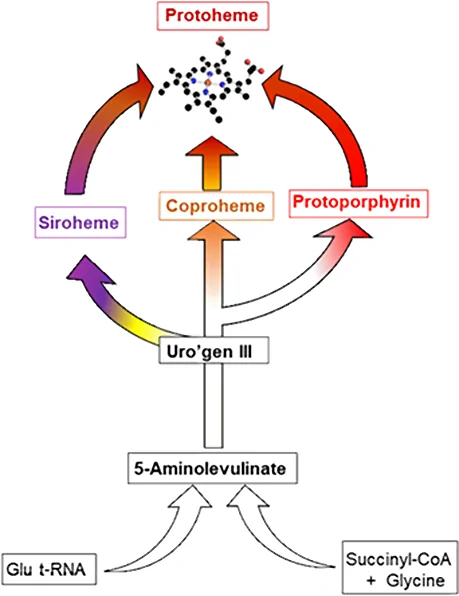

## 1 Introduction

Heme (protoheme IX), an organic cofactor composed of the protoporphyrin IX tetrapyrrole macrocycle with a centrally bound iron atom, is a compound found in a majority of life forms characterized to date. While most organisms can synthesize their own heme,^1^ there are some that possess heme-containing proteins, but do not synthesize their own heme. Among these are several aquatic bacteria,^2^ as well as some pathogenic prokaryotes that are auxotrophic for heme.^3^ Additionally, most parasitic protozoa acquire heme from their host^4^ and members of the phylum Nematoda are heme auxotrophs that acquire heme from their diet or obligate endosymbiotic bacteria.^5,6^ Interestingly, ticks also lack a complete heme biosynthetic pathway and acquire their heme from hemoglobin in their blood meal.^7^

Heme, because of the deep red color it gives to blood and muscle, is most acknowledged for its role in hemoglobin and myoglobin. Even without knowing what heme was, historians through the ages noted that it was essential for life when referring to blood shed in battles or from traumatic injuries. However, heme is much more than just a cofactor for globins. It is a compound that participates in numerous one-electron and redox reactions, functions as a gas carrier, is involved in xenobiotic metabolism, serves as a dietary iron source, is a transcriptional modulator, acts as a biological sensor, functions in RNA metabolism, and is a precursor to a variety of plant accessory pigments and bilirubin in mammals.^1^ Heme homeostasis is crucial for regulating diverse cellular metabolic pathways.^8^ In mammals, defects in the heme biosynthetic pathway may result in pathological disease conditions named porphyrias.^9,10^ In other words, heme is essential for life in the organisms in which it is found and the inability to make or obtain heme may be lethal.

Over the past century researchers have gone from characterizing the chemical nature of heme to identifying biosynthetic pathways, studying the structure and function of the biosynthetic enzymes, and investigating diverse regulatory mechanisms. In the current work a review of the history of research on the biosynthetic pathways to heme is presented. The manuscript is organized so that sections 1 through 6 highlight the elucidation of heme biosynthetic pathways and sections 7 to 10 discuss the characterization and mechanistic understanding of the enzymes responsible for each step. Section 11 highlights a few questions yet to be answered. Realizing that not all readers will have in-depth knowledge of the biosynthetic pathway, Figure 1 presents a model of the classical pathway as it exists in metazoan organisms.

**Figure 1:**
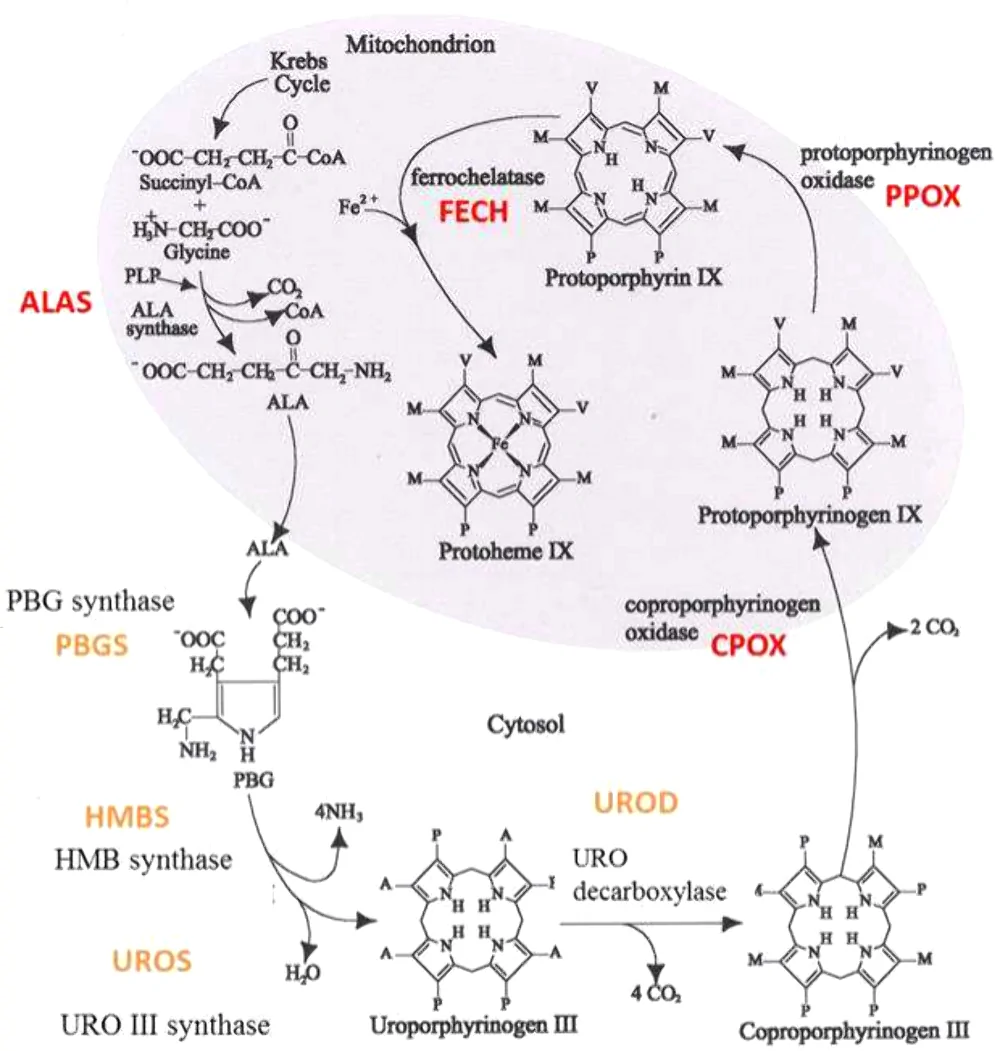
Mammalian heme biosynthetic pathway. Enzymes located in the mitochondrion are shown in red, those in the cytosol are in orange. The gray shaded area represents the mitochondrion including both matrix and intermembrane space.

For a deeper historical look at the early days of research, two early reviews of note are by R. Lemberg and J.W. Legge^11^ and C. Rimington.^12^ These give considerable background and details of early investigations that are infrequently mentioned in more modern reviews. At the time of those reviews, much of the biosynthetic pathway was a black box, so these older documents provide insight into various chemical/theoretical approaches and proposals for heme synthesis. Particularly interesting were models proposed for synthesis of the pyrrole moiety and, later, diverse proposals for synthesis of uroporphyrinogen III. Looking back from the present, following the steps and missteps is as intriguing as reading a good detective story. A more recent review that covers research conducted to elucidate pathways for synthesis of all tetrapyrrole compounds is the one by A.R. Battersby.^13^

Herein no attempt was made to review the history of research on the porphyrias since a number of excellent reviews on this topic are available.^9,10,14,15^ Additionally, there is no attempt to discuss studies on regulatory mechanisms, heme trafficking, heme degradation, or detailed enzyme structure/function since each of these topics represents large numbers of investigations worthy of their own reviews which are cited at appropriate places below (see refs 3 and 16–22 ). Likewise, while fascinating, the complexity of porphyrin and heme synthesis and its compartmentalization and regulation in plants is beyond the scope of the current review. I have attempted to identify and credit those who made critical initial discoveries. But there are excellent studies that are not discussed since such an inclusive effort would have resulted in an impossibly long manuscript and reference list.

## 2 Early Chemistry

The history of research related to porphyrins in biology and chemistry extends back almost two centuries. One might consider the birth of porphyrin research to be the 1840s when techniques employing concentrated sulfuric acid to remove iron from hemoglobin-derived heme (then named haematin) were first published by the Swedish chemist, Jöns Jacob Berzelius,^23^ the German chemist J.J. Scherer^24^ and Dutch chemist G.J. Mulder.^25^ Of some interest, work on haematin was a minor focus for each of these chemists. Berzelius is considered the father of modern chemistry, Scherer is best known for his discovery of lactic acid in blood during pathological conditions, and Mulder best recognized for coining the term “protein”. It would be another two decades before J.L.W. Thudichum^26^ produced, characterized and crystallized the red, fluorescent “iron-free hematin” that he named “cruetine” from the Latin cruor meaning spilled or clotted blood, in contrast to sanguis that referred to circulating blood. Thudichum, who was born and trained in Germany, but immigrated to Britain in 1853, was largely underappreciated during his lifetime, but is now considered to be the father of neurochemistry. He was a master at isolating and characterizing biological compounds, of which cruetine was a minor one for him.^27^

The origin of the word “porphyrin” as a name for iron-free heme may be credited to Felix Hoppe-Seyler. Born Felix Hoppe, he took on the name Seyler from his older sister’s husband who had raised him after being orphaned at the age of 11 years. Hoppe-Seyler is considered by many to be the father of biochemistry (physiological chemistry).^28^ He first named iron-free heme from blood as hematoporphyrin^29^ and metal-free chlorophyll as phylloporphyrin.^30^ The choice of the word porphyrin was an interesting one since it derives from the Greek word porphua/porphy which mean purple. Given that the Greek word for red is eruthos/erythro, it seems more appropriate to have coined the name erythrin, especially since erythro- was not utilized until around 1894 to describe red blood cells as erythrocytes. Perhaps Hoppe-Seyler had been inspired by classical Roman statues and sarcophagi made out of the rare and valuable stone red porphy, or by the Greek mythological giant Porphyrion. Whatever his rationale, our metal-free, red-colored macrocyclic tetrapyrroles are somewhat misnamed via Greek as porphyrins. An interesting aside concerning the naming of tetrapyrrole compounds is that J.J. Berzelius, who coined the name biliverdin for the green pigment, preferred the more accurate term bilifulvin (yellow) rather than bilirubin for the yellow pigment,^23^ but that preference was not adopted by others.

Regardless of the possible inappropriateness of the name porphyrin, it was not immediately embraced by all researchers at that time leading to descriptions of the urine derived pigments urofuscohaematin and urorubohaematin (uro- from the Greek *ouron* meaning urine) by Baumstark^31^ and later urospectrin by Saillet.^32^ This creates some confusion in the early literature. However, by the turn of the century the term porphyrin was well accepted. Despite Baumstark’s decision to not utilize the term porphyrin, he was ahead of his time by proposing that porphyrins in clinical samples originated from heme precursor molecules and not breakdown products as was generally believed at that time.^33^

The late 19th and early 20th century research into the chemistry of tetrapyrroles was conducted largely by German born and trained chemists using materials obtained from clinical specimens. Most research was connected to the studies of porphyrias. Reviewing the immense body of porphyria-related work is outside the focus of the current work, but it has been frequently and expertly reviewed by others.^9,12,14,15,34^ The identification of the actual porphyrins present in the samples was hampered by a lack of uniform methodologies and assigned names until early 20th century publications by Willstatter,^35,36^ Küster^37^ and Fisher.^38^ At this time the names uroporphyrin, coproporphyrin (from the Greek *kopros*- reflecting the feces as the source), and protoporphyrin came into general usage. In the 1920s Hans Fischer, who was trained in both chemistry and medicine, and co-workers experimentally determined the structure of protoporphyrin IX. Previously Küster^37^ had proposed what was later shown to be the correct skeletal formula of heme, but at the time it was rejected by others, including Fischer. Fischer also determined the structures of the I and III isomers of copro- and uroporphyrin. His work revealed experimentally for the first time the beautifully complex tetrapyrrolic structure of porphyrins (Figure 2). For this copious volume of work, Fischer was awarded the Nobel Prize in 1930.^39^ His research approaches relied upon porphyrins obtained from human material and these were greatly aided by a laboratory worker named Mathias Petry who had “congenital hematoporphyria” and excreted large quantities of porphyrins in his urine (a liter of his urine yielded 80 mg of uroporphyrin). This proved to be extremely valuable to Fischer’s efforts. Petry was sought after by competing groups, and it was said by Fischer that Petry came to him in Munich because he preferred the famous Munich beer. Upon Petry’s death in 1925 Fischer conducted a chemicophysiological autopsy that he published under the title of “Porphyrinurie”.^40^ During the war Fischer’s lab was destroyed and, tragically, he committed suicide in 1945. Much of Fischer’s work is reviewed by Lemberg and Legge^11^ and With.^34^

**Figure 2:**
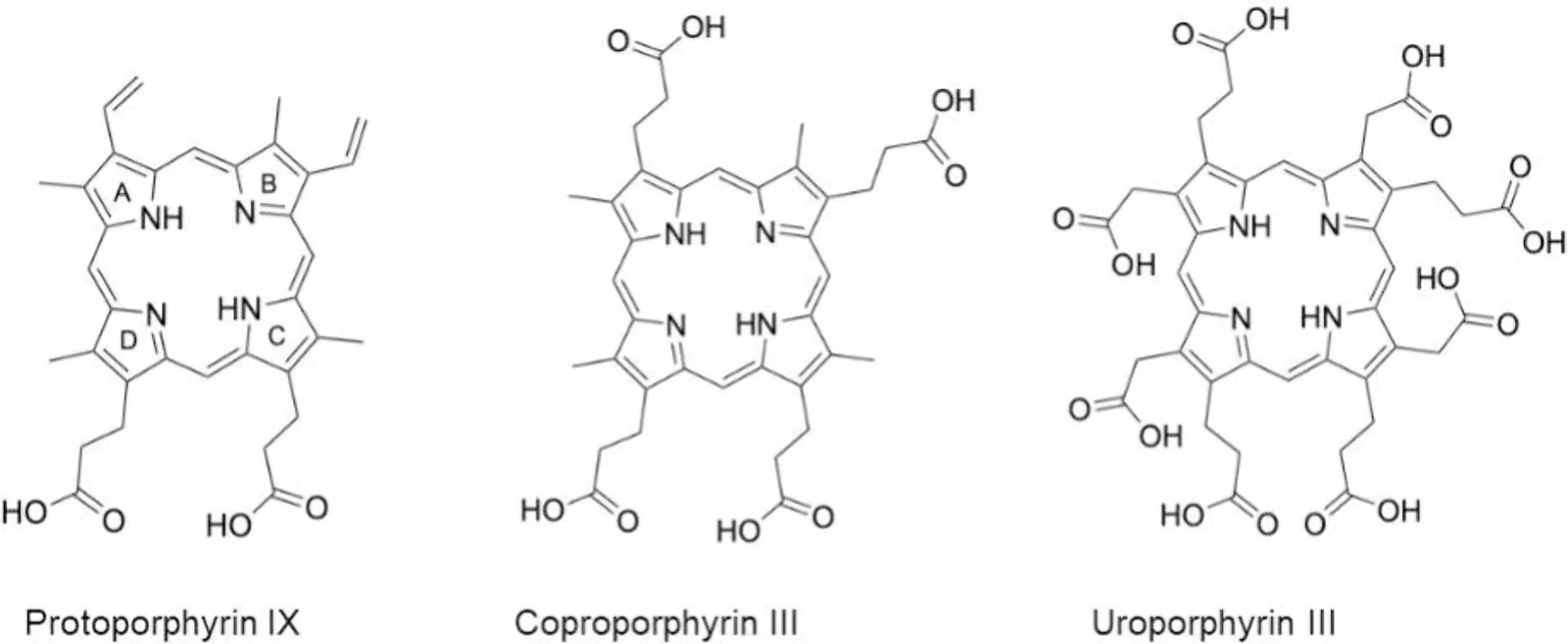
Chemical structures of protoporphyrin IX, coproporphyrin III, and uroporphyrin III determined by Hans Fisher. Note that the pyrrole ring D is inverted relative to the other three pyrrole rings. The I isomer of coproporphyrin and uroporphyrin do not have the D ring inverted.

## 3 Elucidation of the Heme Biosynthetic Pathway

### 3.1 Identification of Pathway Precursors

With the determination of the structure of porphyrins obtained from biological samples, rapid progress might have been expected in investigating the biochemical synthetic pathway to heme. However, this was not the case. While significant efforts were put forward to clinically characterize the porphyrias, it would be another two decades before the first published research examining heme biosynthesis. The simple reason for this was that experimental techniques were not available at that time to trace individual carbons through a putative pathway. This became possible only after isotopic nitrogen and carbon became available to researchers. David Shemin, a newly appointed assistant professor in 1945, and Professor David Rittenberg at Columbia University were first to focus on the biochemistry of the pathway.^41,42^ Columbia was a propitious posting for Shemin since Rittenberg, along with Rudolph Schoenheimer, was a pioneer in the use of heavy isotopes to study metabolism.^43^ In what is now considered a classic experiment of the time, Shemin ingested 66 g of ^15^N glycine, then collected his blood over a period of time, isolated the heme from his hemoglobin, and determined that the nitrogen of glycine was a precursor to protoporphyrin.^44^ Additional studies confirming this were carried out in rats.^45^ This was an important discovery since it eliminated a possibility then considered that proline was the pyrrole precursor. Their experiments also allowed them to determine that the average life span of a red blood cell was 127 days.^46^ Following up on these discoveries they achieved the first in vitro synthesis of heme from glycine.^47^

The fate of glycine carbon atoms in the synthesis of heme, while frequently attributed to Shemin, was the work of several groups, all of whom employed isotopic labeling approaches. Reading through the original publications is as much about the chemistry and technique development as it is about final results. Kurt Altman’s group at the University of Rochester, New York, first published in 1948 that the α-CH_2_ of glycine is incorporated into protoporphyrin in rats.^48^ They proposed glycine was used intact to synthesize a pyrrole compound and only after that the glycine carboxyl carbon would be removed. Moises Grinstein and collaborators at Washington University demonstrated that the carboxyl carbon of glycine was not incorporated into protoporphyrin IX, or coproporphyrin I, but was incorporated into the globin chain in dogs.^49,50^ Neither the Altman, nor Grinstein groups carried out an analysis to identify what portion of the porphyrin macrocycle was labeled, nor if the glycine ^14^C carboxyl group found in globin chains was glycine or derived from other amino acids.

Identification of the position of the isotopic label in the macrocycle would be relatively trivial with computer-based NMR and/or mass spectrometry available today. However, these approaches were not available in the middle of the 20th century, so the only approach was to isolate the heme and develop chemical degradation schemes for the macrocyle to determine the position of the isotope label. That daunting task was undertaken by Helen Muir in Albert Neuberger’s laboratory at the National Institute for Medical Research in London. Prior to entering the heme research field, Bavarian-born Neuberger, who had earned an MD *summa cum lauda* and emigrated to England in 1932, had a decade of research on glycoproteins, protein metabolism and amino acids.^51^ He had employed isotopic labeling to approach experimental questions and his interest in porphyrins was stimulated by the publications of Shemin and Rittenberg. Muir and Neuberger developed a small-scale protocol for partial degradation of porphyrin to determine the position of the isotopic label in the macrocycle.^52^ They then employed that new approach with isotopically labeled heme from rabbits.^53^ They found that there are twice as many carbon atoms from glycine as there are nitrogen atoms. All four porphyrin nitrogen atoms are labeled by glycine and four glycine-derived carbons contribute to the methene bridges and four more are in the pyrrole rings. This led them to propose that 2 glycine molecules were fused to make either hydroxy-aspartic acid or aminofumarate, which then condensed with 2 molecules of α-ketoglutarate (α-KG) to form a pyrrole.^53^

Just as Muir and Neuberger had done, Jonathan Wittenberg in Shemin’s lab^54^ developed techniques to degrade relatively small amounts of isolated heme. In these experiments they chemically degraded heme isolated from the blood of ducks that had been injected with ^15^N glycine, and from a woman with polycythemia who had ingested 48 g of ^15^N glycine over a two-day period. Their data showed that all four pyrrole nitrogens originated from glycine. Then Shemin and his students Norman Radin and Wittenberg^55–58^ utilized ^15^N glycine, ^14^C glycine and ^14^C acetate in a series of experiments employing *in vivo* labeling of heme to demonstrate that the α-CH_2_ carbon atom from glycine is present in protoporphyrin as a pyrrole α-carbon as well as in the methene bridges. These findings agreed with those of Muir and Neuberger discussed above. Using ^14^C succinate, Shemin and Kumin^59^ demonstrated that “activated” succinate contributed the remainder of carbon atoms to protoporphyrin. They postulated that this activated compound may be succinyl CoA, a compound that would not be identified until a year later.^60^ This proposal was further supported by data from Wriston, Lack and Shemin.^61^

Interestingly the model for synthesis of the pyrrole proposed by Shemin and Kumin^59^ had two molecules of “activated” succinate reacting with one molecule of glycine to form a “common pyrrolic precursor”. The formation of the tetrapyrrole would require four of these along with four carbon molecules from a glycine-derived compound that would connect the four rings via the methine bridges. Thus, three different research groups had come up with three distinct models for formation of a pyrrolic intermediate from glycine^48,52,59^ and all were incorrect. None had guessed the nature of the true precursor that was the intermediate between glycine and succinyl CoA, and the pyrrole compound. Evidence that none of these models was correct was provided by the characterization of porphobilinogen (PBG) as a substituted pyrrole (2-aminomethyl-4–2’-carboxyethyl-3-carboxymethylpyrrole) (Figure 3) by Cookson and Rimington.^62,63^

**Figure 3:**
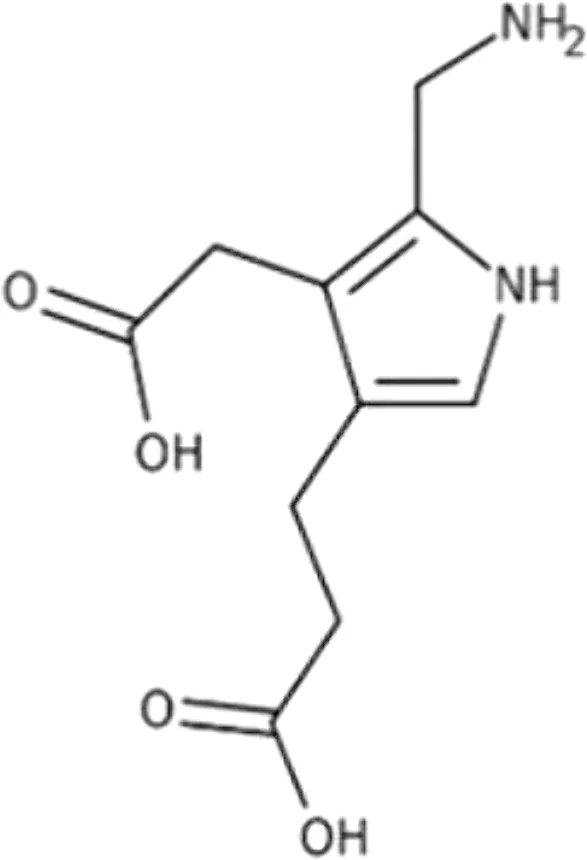
Chemical structure of porphobilinogen.

### 3.2 Identification of ALA and PBG as Pathway Intermediates

Claude Rimington, who had a long, varied and significant history in biomedical science following his graduation from Cambridge, had established a strong internationally recognized group focused on pyrrole research at the University College Hospital Medical School in London following WWII.^64^ PBG had initially been reported to be in urine of porphyric individuals by Waldenström^65^ and the compound was later isolated from urine of a patient with “acute porphyria” by Rimington’s chemistry colleague R.G. Westall.^66^ The chemical characterization of PBG was probably largely the work of Rimington’s colleague G.H. Cookson, an organic chemist. The structure of PBG was quite similar to the “common pyrrole precursor” previously proposed by Shemin and Kumin,^59^ except that PBG possessed a terminal amino group adjacent to what would become the methine bridge carbon.

Support for PBG being a bonafide intermediate in the pathway came from work by Rimington’s first assistant Elizabeth Dresel and Australian colleague John Falk.^67,68^ (Falk may best be remembered for his book, *Porphyrins and Metalloporphyrins*, published in 1964, which was the first effort to present in one volume the chemistry and physics of tetrapyrrole compounds.^69^). They demonstrated that PBG was an effective precursor to uro-, copro- and protoporphyrin in hemolysates of avian red cells. Bogorad and Granick,^70^ at the Rockefeller Institute for Medical Research, demonstrated the same in the green alga *Chlorella*. So, a role for PBG in the biosynthetic pathway to protoporphyrin was clear, but the synthetic route to PBG from glycine and the “activated” succinate remained uncharacterized.

The structure of PBG with its 2-aminomethyl side chain and the fact that its precursor compounds were glycine and succinyl CoA, hinted at its synthetic route. Shemin, with his postdoc Charlotte Russell, proposed that the actual precursor to PBG was 5-aminolevulinic acid (ALA) that was formed from glycine and succinyl CoA.^71^ Shemin initially considered that formation of ALA from glycine and “activated succinate” may be a two-step process; a condensation of the glycine with succinate to form α-amino-β-ketoadipate, followed by a decarboxylation (of what had been the glycine carboxyl carbon) to yield ALA. Neuberger and Scott^72^ verified that ALA was a precursor to protoporphyrin, while Dresel and Falk^67,68,73,74^ demonstrated the conversion of ALA to PBG, uro-, copro- and protoporphyrin. Shemin’s group demonstrated that ALA could serve as a precursor to protoporphyrin in duck red cell hemolysates^75^ and proposed that two molecules of ALA were condensed via a Knorr type mechanism to form PBG (Figure 4). This scheme would support the distribution of glycine-derived carbon atoms and account for the glycine carbon to nitrogen stoichiometry of 2 to 1 found in protoporphyrin. Neuberger and colleagues demonstrated the enzymatic condensation of ALA to form PBG^76,77^ by a partially purified fraction from ox liver. They named the enzyme ALA dehydrase (now named PBG synthase). Such an *in vitro* reaction was also reported independently by Granick in chicken erythrocytes.^78^

**Figure 4:**
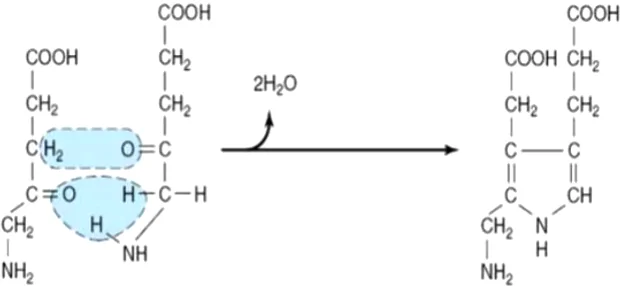
Knorr condensation of two ALA molecules to form PBG.

Thus, by 1955 there was general agreement among researchers that the initial steps to synthesize heme in animals started with glycine and “activated” succinate (succinyl CoA) to form ALA and then two molecules of ALA were condensed to form PBG. Schiffmann and Shemin^79^ demonstrated that the labeling pattern in protoporphyrin from ^14^C ALA corresponded with what would be expected from the proposed mechanism (Figure 5).

**Figure 5:**
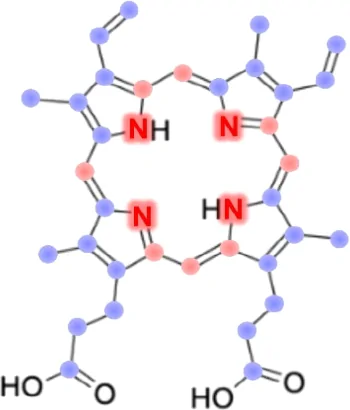
Protoporphyrin IX. Atoms derived from glycine are shown in red and those from succinyl CoA are in blue.

Independently, Berlin, Neuberger and Scott^80,81^ administered ^15^N or ^14^C labeled ALA to human subjects, recovered heme from red cells and demonstrated that in humans, ALA was a precursor to protoporphyrin and essentially no other compounds. Interestingly, it was noted that the human subjects in these experiments developed dose dependent photosensitivity. Thus, ALA-based photodynamic therapy as implemented in the 1990s^82^ had its roots in this somewhat casual observation.

Shemin proposed that ALA may participate in what he called the succinate-glycine cycle.^83^ This was supported by the observation that labeled ALA contributes to cellular succinate and the ureido group of guanine in duck red blood cells. This was also reported later in facultative photosynthetic bacterium *Rhodospirillum rubrum* by K. Shigesada.^84^ More recently M. Petricek et al.^85^ found that ALA is utilized as the precursor for antibiotic production in *Streptomyces nodosus.* However, data supporting a significant role for ALA as a precursor to other cellular components is currently lacking within the metazoa.

Neuberger’s group working with freeze-dried chicken erythrocyte fractions^86,87^ and Shemin’s laboratory with *Rhodobacter* (then *Rhodopseudomonas*) *sphaeroides*,^88,89^ characterized the enzymatic nature of ALA synthesis (at that time named ALA synthetase). This synthesis utilized glycine and succinyl CoA and was stimulated by pyridoxyl phosphate (Figure 6). The product of the reaction was shown to be ALA.^89^

**Figure 6:**
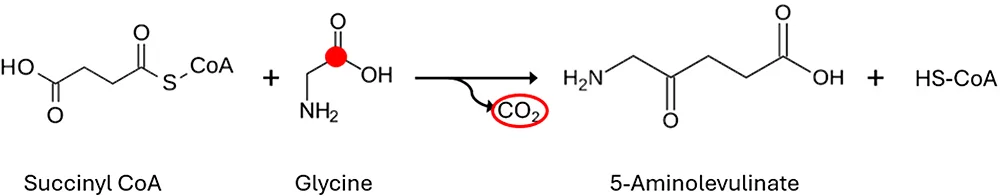
Formation of ALA from glycine and succinyl CoA as catalyzed by ALA synthase. The glycine carbon that is not incorporated into ALA is shown in red.

An interesting historical perspective on these studies is presented by Shemin.^90^ It is of note that while early studies largely involved tissue samples from humans, and avian (mainly duck) blood, significant progress occurred following the adoption of the bacterium *R. sphaeroides* as a model organism. June Lascelles,^91^ then at the University of Oxford, examined in considerable detail the synthesis of porphyrins by cell suspensions of *R. sphaeroides*.^92,93^ She was encouraged by a year spent in 1956 with the acclaimed microbial physiologist C.B. van Neil at Stanford University Hopkins Marine Station to examine tetrapyrrrole production by this member of the Athiorhodaceae, which are facultative photosynthetic bacteria.^92,93^ Shemin acknowledged that her work had encouraged him to utilize *R. sphaeroides* in his studies. *R. sphaeroides* also became a workhorse for studies of tetrapyrrole synthesis in the 1960s by George Tait with Neuberger’s lab (see below). Selection of this organism for the study of heme biosynthesis was true serendipity since only the alpha-proteobacteria, of which *R. sphaeroides* is a member, possess ALA synthase (ALAS). It would be two more decades before it was found that all other bacteria lack ALAS and utilize an alternative pathway to synthesize ALA.

### 3.3 Synthesis of Uroporphyrinogen III

The mechanism by which four molecules of PBG polymerize to form the III isomer of uroporphyrinogen remained the object of considerable speculation. Interesting discussion of this question can be found in the older reviews by Battersby and McDonald^94^ Frydman et al.,^95^ and Bullock et al.^96^ Various schemes were proposed including the formation of a tripyrrylmethane that breaks down into a dipyrrylmethane. Dependent upon the position of bond breakage, this could yield two different dipyrrylmethane molecules. These would then combine to form the III isomer tetrapyrrole.^75^ Another proposal went via a “T” tripyrrylmethane intermediate (Figure 7).^70^ The “T” proposal gathered some interest from studies on the molecule prodigiosin, a red pigment synthesized by the bacterium *Serratia marcescens*.^97^ At that time this molecule was believed to be a tripyrrylmethene whose structure was very similar to the one proposed by Bogorad and Granick as an intermediate in the synthesis of uroporphyrin. In part because of this similarity, the biosynthesis of prodigiosin garnered attention as possibly being derived from ALA.^98^ However, both the proposed “T” structure and the tripyrrylmethane structure proposed for prodigiosin were incorrect. Additionally it was eventually determined that prodigiosin was synthesized from l-proline and l-serine, and not from ALA.^99^

**Figure 7:**
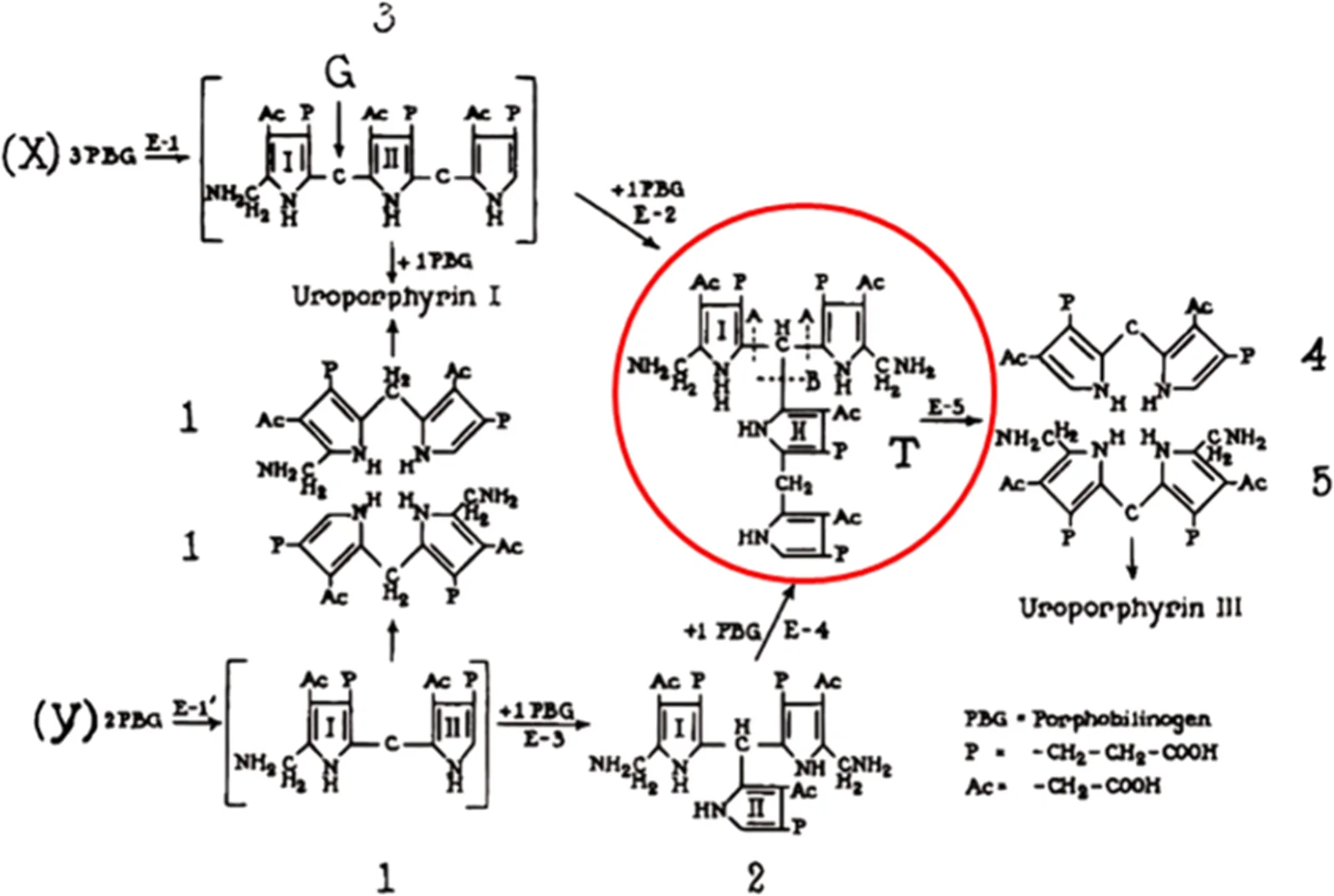
″T” model for generation of uroporphyrinogen III. This figure is modified from that found in Bogorad and Granick, 1953. The red circle highlights the “T” structure formed from the condensation of two dipyrrylmethane molecules.

The schemes of Shemin, Russell and Abramsky^75^ and Bogorad and Granick^70^ for the formation of uroporphyrinogen III (uro’gen III) were rejected by Bullock et al.^96^ and Lockwood and Benson^100^ based upon chemical principles. Cookson and Rimington^63^ proposed a model where the first two PBG molecules formed a symmetrical dipyrromethene onto which two additional PBG molecules would be added. This scheme was modified slightly by Bullock et al.^96^ and later by Frydman’s group.^101,102^ However, this scheme proved to be inaccurate. Coming close was Pamela Cornford, a doctoral student advised by Rimington, who proposed synthesis of a linear tetrapyrrole that had its last pyrrole ring added in an inverse manner to form the III isomer.^103^ Overall, over two dozen models were proposed. Most accurate was the one proposed by Mathewson and Corwin^104^ who proposed flipping of the D ring during closure of the linear tetrapyrrole. However, data supporting an enzyme mechanism for the formation of uro’gen III from PBG was not to come for another two decades (see below, Figure 17). Batlle and Rossetti^105^ present an overview of available data and thoughts on this topic in the late 1970s, and excellent in-depth reviews of these models are presented by F.J. Leeper^106^ and P. M. Jordan.^107^

An initial step toward defining the synthesis of uro’gen III was made by Lawrence Bogorad^108^ while a postdoctoral fellow in Samuel Granick’s laboratory at the Rockefeller Institute for Medical Research. As part of their studies on porphyrin synthesis in *Chlorella* they demonstrated that PBG could be converted to protoporphyrin IX by cell-free extracts, but if they first heat treated the extracts to 55 °C, only uroporphyrin I was produced.^70^ This was the first clue that conversion of PBG to uroporphyrin III may be a two-step process which required a heat stable enzyme to form the I isomer and a second heat labile enzyme necessary for formation of the III isomer.

Later, Bogorad, then an assistant professor at the University of Chicago, made the crucial observation that in spinach leaf acetone extract, pathway intermediates were porphyrinogens and not porphyrins.^109^ The initial clue was that whatever the intermediates were, they were colorless, in contrast to the brightly colored and fluorescent porphyrins. Porphyrins are fully conjugated macrocycles (that is, they have a maximum number of double bonds) composed of four pyrroles connected by bridging methene carbons (Figure 8) They are rigid, planar molecules that have intense orange to red color and are highly fluorescent. Porphyrinogens, on the other hand, are not fully conjugated having double bonds only in the individual pyrrole rings and not with the bridging carbons connecting the rings. Thus, they are flexible molecules that may assume diverse conformations by rotating about the bridging methane bonds. They are colorless, nonfluorescent compounds. However, they are highly reactive structures that readily undergo a six-electron oxidation in air to porphyrins.

**Figure 8:**
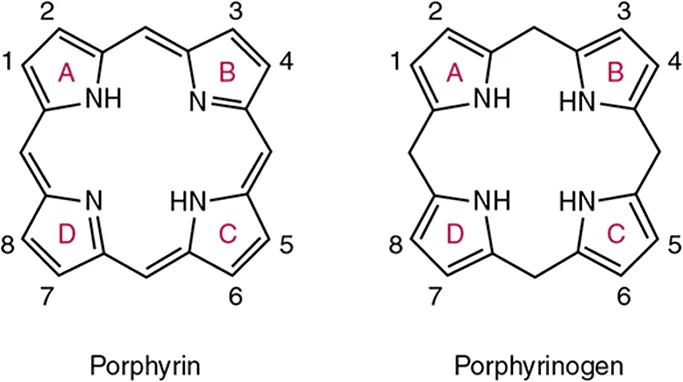
Structures of porphyrin vs porphyrinogen molecules. The pyrrole rings are labeled A, B, C, and D as shown in Figure 2. Also shown is the accepted convention to number the positions of the side chains as 1 through 8. For uroporphyrin­(ogen) III, 1, 3, 5, and 8 are acetate side chains, and 2, 4, 6, and 7 are propionate side chains.

The importance of the discovery that porphyrinogens, and not porphyrins, were biosynthetic intermediates, should not be understated. It was significant since uro- and coproporphyrin, which had been demonstrated by multiple research group to arise from ALA or PBG, had never been found to serve as precursors to heme. Shemin had reported earlier without data that coproporphyrin III is not converted to protoporphyrin in duck erythrocyte hemolysate,^75^ and Dresel and Falk^110^ presented data showing that porphyrins were not intermediates to heme. It had even been suggested that uroporphyrin or coproporphyrin were breakdown products of the actual pathway intermediates. Neve, Labbe and Aldrich at the University of Oregon Medical School^111^ provided additional support for Bogorad’s discovery when they demonstrated that uro’gen III is converted to protoporphyrin IX and heme by lysed duck erythrocytes and that uro’gen III effectively competes with isotopically labeled ALA in the production of heme. Bogorad^112^ further demonstrated in *Chlorella* extracts that only uro’gen I or III, and not uroporphyrin I or III, were utilized in the synthesis of coproporphyrinogen (copro’gen) I or III.

Bogorad examined the conversion of PBG to uro’gen III in detail using extracts of *Chlorella* and wheat germ in a series of papers.^112–114^ He reported an enzyme that he named PBG deaminase was responsible for the condensation of four molecules of PBG to form uro’gen I. Uro’gen III was only formed in the presence of this enzyme plus an additional enzyme he named uro’gen isomerase (Figure 9). Significantly, Bogorad noted that “the isomerase participates in the synthesis of uroporphyrinogen III prior to the cyclization of the tetrapyrrole. . .”.^114^ Similar studies were done with *R. sphaeroides* by D. S. Hoare, a colleague of Rimington at University College Hospital Medical School in London^115^ and their data were consistent with those of Bogorad.

**Figure 9:**
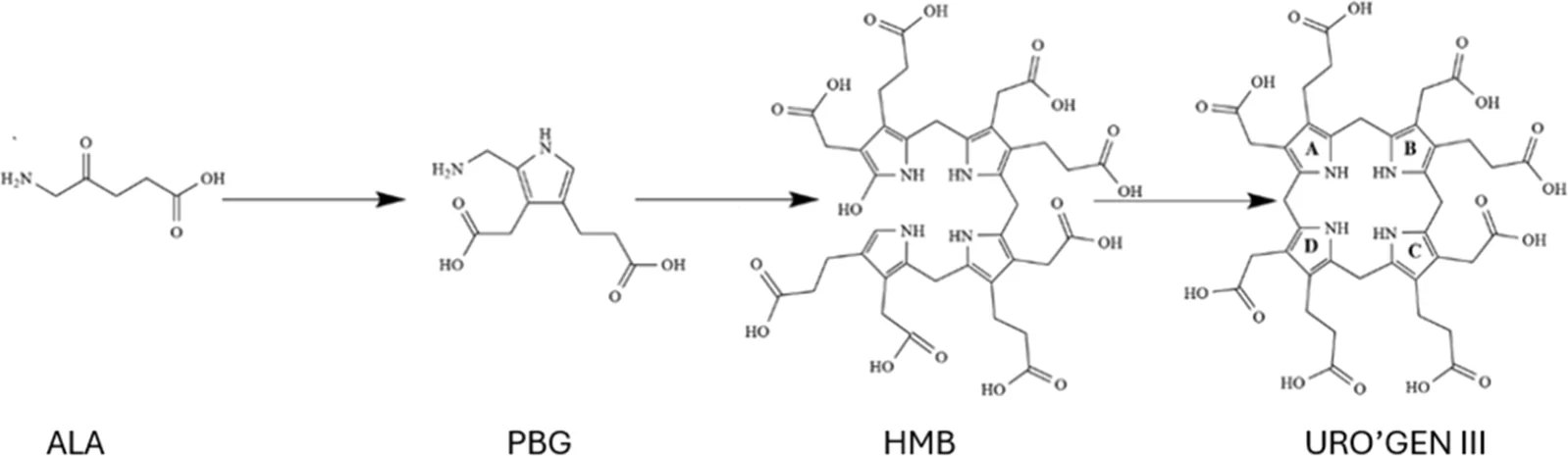
Synthesis of uroporphyrinogen III from ALA. This segment of the tetrapyrrole biosynthetic pathway is conserved in all organisms. The currently accepted names for the enzymes involved are PBG synthase, HMB synthase, and uro’gen synthase.

### 3.4 Decarboxylation of Uro’gen III to Proto’gen IX

In the same study Heath and Hoare also demonstrated uro’gen decarboxylase activity that converted uro’gen I and III into copro’gen I and III, respectively, by acetone-dried powder from *R. sphaeroides.* These observations settled the ongoing question as to why putative pathway intermediates, uroporphyrin and coproporphyrin, did not serve as precursors to protoporphyrin in various cell homogenates (see ref 12). In a series of papers in the Journal of Biological Chemistry, Granick^116^ explored the experimental fractionation of the heme biosynthetic pathway in hemolyzed rabbit red cells.^117–119^ His group validated previous reports that only porphyrinogens, and not porphyrins, were biosynthetic intermediates, and they demonstrated that a fractionated cellular preparation decarboxylated uro’gen I and uro’gen III to copro’gen I and III, respectively.^119^ The enzyme, named uroporphyrinogen decarboxylase,^118^ did not use uroporphyrin I or III as a substrate (Figure 10). They noted that copro’gen III, but not copro’gen I, was converted by “insoluble components” into protoporphyrin.

**Figure 10:**
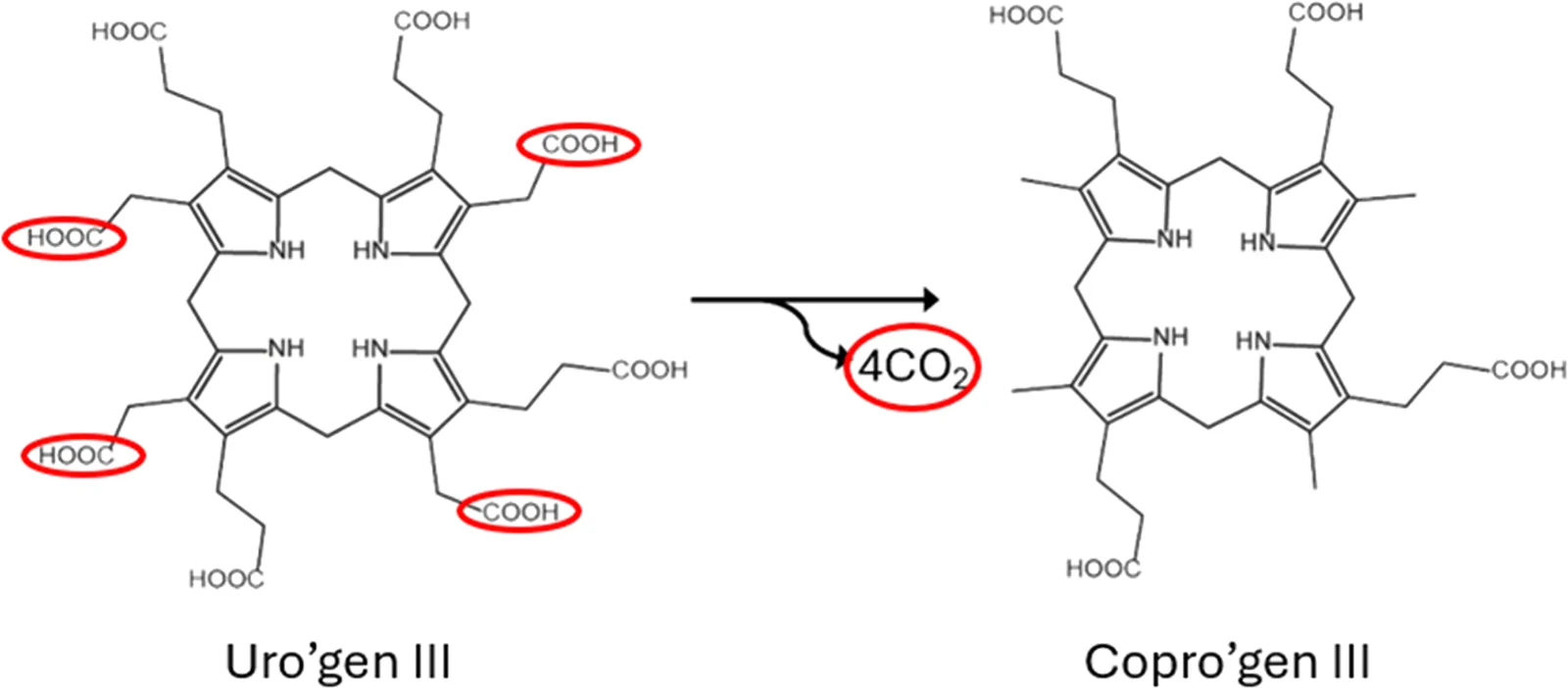
Decarboxylation of uro’gen III to copro’gen III. Carboxyl groups that are removed in this reaction by uro’gen decarboxylase are outlined in red.

### 3.5 Terminal Steps

Based upon studies with crude cell extracts, it was proposed independently by J.R. Klein^120^ and M. M. Wintrobe^121,122^ that the final step of heme synthesis was insertion of iron into protoporphyrin IX and that this was an enzyme-catalyzed reaction. However, as noted by Krueger at al., “the relationship between the amount of heme formed and protein content. . .does not allow certain assignment of the activity to an enzyme”.^120^ While the data from Wintrobe’s studies clearly demonstrated that iron insertion into protoporphyrin, but not coproporphyrin or uroporphyrin, was catalyzed by avian cell extracts, it was three publications arising from Robert Labbe’s laboratory at the University of Washington that clearly defined this step and reported it as a mitochondrially located process.^123–125^ The enzyme utilizes protoporphyrin IX, but not coproporphyrin III, and ferrous iron. The name given to this enzyme was ferrochelatase or heme synthetase.

In 1961 Sano and Granick demonstrated the conversion of copro’gen III to protoporphyrinogen (proto’gen) IX by a protein fraction obtained from mitochondria.^126^ The name given the enzyme was coproporphyrinogenase or copro’gen oxidase. Neither copro’gen I nor coproporphyrin served as substrates for this enzyme and oxygen was required for the reaction. They noted that the product was proto’gen, but that in the presence of a freeze-dried mitochondria fraction, the porphyrinogen was converted to protoporphyrin. They hypothesized that a mitochondrial enzyme existed to catalyze this oxidation. However, it would be over a decade before this would be experimentally demonstrated by others (below).

Thus, by 1961 the research groups of Shemin, Neuberger, Falk, Granick, Bogorad, Rimington and Labbe had identified and validated in a variety of cell types all but the penultimate steps of heme synthesis. They had presented data showing that heme synthesis originated with ALA production from glycine + succinyl CoA, followed by condensation of two ALA molecules to form PBG. Four PBG molecules are then polymerized via a two-step process to yield uro’gen III, which is subsequently decarboxylated to copro’gen III then protoporphyrinogen IX. Following oxidation to protoporphyrin by an uncharacterized oxidase, ferrous iron is inserted to form heme.

It was noted that ferrochelatase catalyzed the insertion of ferrous, but not ferric iron. Ferrous iron rapidly oxidizes under physiological conditions to ferric iron and iron transport and storage compounds are ferric iron, so a ferric reductase to support heme synthesis is essential. The ability of mitochondria to reduce ferric iron to ferrous iron for utilization by ferrochelatase was demonstrated in 1972 by Owen Jones research group at the University of Bristol.^127^ Jones had previously demonstrated that ferrochelatase was located in the mitochondrial inner membrane.^128^ He found that a functional respiratory chain was necessary for this activity. While this observation was key and hinted at intracellular iron metabolism, there was essentially no advancement in the field of “ferric reductase” because of the cumbersome and indirect assay for measuring ferric iron reduction. Characterization of ferric reductase activity, both for heme biosynthesis as well as all other cellular iron-based processes, expanded greatly after the introduction of ferrozine as a chromophoric ferrous iron trap by Harry Dailey, then a graduate student in Lascelles laboratory at UCLA.^129^ This reagent allows for direct spectroscopic observation of ferric iron reduction to ferrous iron in biological systems.

There was a bump in the road for ferrochelatase when R. Tokunaga and S. Sano,^130^ and R .J. Kassner and H. Walchak^131^ reported that iron insertion into protoporphyrin could occur nonenzymatically under *in vitro* ferrochelatase assay conditions then in use. The clear demonstration of the enzymatic nature of the reaction came with the isolation and characterization of a mutant strain of the bacterium *Spirillum itersonii* that lacked ferrochelatase activity and was a heme auxotroph.^132^ Spontaneous revertants regained activity and no longer required heme for growth. Additional support then came from the isolation of a *Saccharomyces cerevisiae* mutant that lacked ferrochelatase activity.^133^

The last step to be experimentally demonstrated was the penultimate step in the pathway; the oxidation of proto’gen IX to protoporphyrin IX. These data were obtained by R. Poulson and W. J. Polglase at the University of British Columbia^134^ who partially purified the enzyme responsible for oxidation from *S. cerevisiae*. The activity was mitochondrially located and the enzyme was named protoporphyrinogen oxidase. The enzyme utilizes proto’gen IX, but not uro’gen I or III, or copro’gen I or III, and requires molecular oxygen for the oxidation. Proto’gen oxidase activity was demonstrated to be distinct from coproporphyrinogenase^135^ as had been suggested earlier by Sano and Granick.^126^ Thus, by the late 1970s all steps in the metazoan heme synthesis pathway had been identified.

### 3.6 The 5-Carbon Pathway to ALA

In spite of the best efforts by numerous research groups, ALA synthase (ALAS) was never found in plants. The first clear evidence for an alternate pathway to ALA in plants came from Samuel Beale, then a postdoctoral fellow in the lab of Paul Castelfranco at the University of California, Davis. He demonstrated that glutamate and not succinate or glycine was a precursor to ALA.^136,137^ Later Beale, then in Granick’s lab at the Rockefeller, found that in plants the five-carbon skeleton of glutamate was incorporated intact into ALA and that glutamate, but not glycine, was a precursor to ALA in *Chlorella*.^138^ Exactly how a 5-carbon precursor was converted into ALA was the subject of speculation.^138^ One popular suggestion at that time revolved around 4,5-dioxovalerate (DOVA) as a putative intermediate that was transaminated to form ALA. James Lohr and Herbert Friedmann^139^ demonstrated that in *Zea mays* leaf extracts there are two enzymes that could catalyze the conversion of glutamate to ALA. Here they found an NADH-dependent reduction of glutamate to DOVA followed by a transamination to yield ALA. The enzyme responsible for this transamination is alanine:4,5-dioxovaterate transaminase (now named alanine-glyoxylate aminotransferase (AGXT2)). AGXT2 is a highly promiscuous transaminase (see ref 140) that has been shown to irreversibly transaminate DOVA to form ALA.^141^ Neuberger and Turner^142^ reported that *R. sphaeroides* possessed DOVA transaminase, however, Jon Takemoto’s laboratory at Utah State University reported that a mutant of *R. sphaeroides* which lacked ALAS activity, but possessed the transaminase activity, was unable to grow without exogeneously supplied ALA.^143^ Additionally, Foley and Beale^144^ presented evidence illustrating that this enzyme is widely distributed in nature and not unique to plants. Thus, a role for DOVA as a precursor to ALA in plants lacks compelling support.

A second proposed alternative was one that involved formation of glutamate-1-semialdehyde (GSA) from glutamate. Evidence in support of this came from the characterization of a GSA aminotransferase by Simon Gough, then in Gamini Kannangara’s group at Carlsberg.^145^ Defining this as a potential intermediate was problematic and subject to question by some since GSA is highly labile (see ref 107). Experimental data from a number of organisms demonstrated that multiple cellular fractions were required for the synthesis of ALA from glutamate.^146–152^ Synthesis of ALA by cellular fractions required the presence of ATP, magnesium, and NADPH. It was proposed that glutamate was “activated” to a form that would then be converted to GSA and this was then transaminated to ALA. The nature of the “activated” glutamate was the key missing factor. One possibility was that glutamate was activated by a kinase to form glutamate-1-phosphate and then converted to GSA.^145^ This proposal was abandoned when it was found in *Chlamydomonas* that an RNA moiety was a necessary component.^153^ Similar results were reported for *Chlorella vulgaris* where it was shown that a tRNA-containing fraction from *C. vulgaris*, but not *E. coli*, supported ALA production in cell extracts that had previously been treated with RNase.^151^ Schön et al.,^154^ then characterized the necessary component as a chloroplast glutamyl-tRNA. Thus, synthesis of ALA in plants was found to require glutamate, a specific glutamyl-tRNA, glutamyl-tRNA synthetase, glutamyl-tRNA reductase, and GSA aminomutase (aminotransferase) (Figure 11) (see ref 155). The identification of a tRNA as a precursor for ALA was not anticipated given the canonical role of tRNAs in protein synthesis. In contrast to the synthesis of ALA via ALAS, which is referred to as the C-4 pathway, the glutamyl-tRNA-based pathway to ALA is referred to as the 5-C pathway.

**Figure 11:**
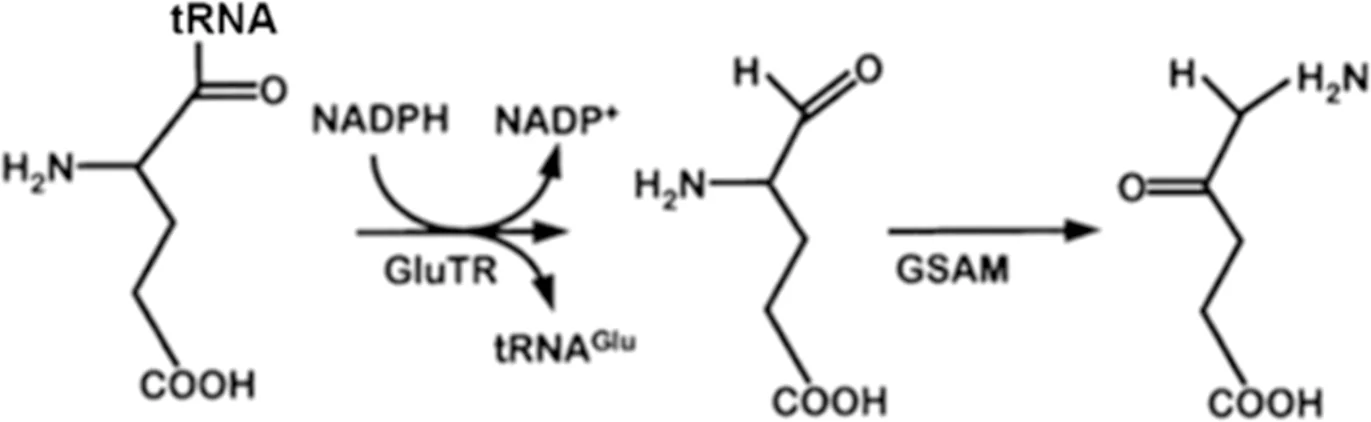
C-5 path to form ALA from glutamate.

Returning to consideration of DOVA as a potential precursor to ALA in nonplant tissues, it was shown by James Kushner and Bruce Burnham’s group at the University of Utah that the mitochondrially located enzyme AGXT2 efficiently catalyzes the transamination of 4,5-dioxovaterate to ALA, using alanine as an amino group donor *in vitro*.^141^ Evidence was presented that demonstrated exogenously supplied DOVA was incorporated into ALA and heme when provided to intact, respiring rat hepatocyte suspensions^156^ and to living rats.^157,158^ In hepatocyte suspensions, the incorporation of DOVA (in the presence of alanine) into porphyrin was linear for a 2 h period. When labeled ^14^C-labeled DOVA was injected into rats, label was recovered in excreted ALA, and in hepatic and erythroid heme at levels similar to those found with labeled glycine. It was noted also that erythroid heme was labeled with DOVA to a higher extent than with labeled ALA. This was the inverse of what was found with hepatic heme. Overall, the data demonstrated that DOVA can serve as a precursor to ALA and heme assuming that DOVA is present in the mitochondria of cells. However, this line of investigation has not been pursued.

## 4 Pathway Adjustments for Anaerobic Life

From limited studies with bacteria, it was assumed that they synthesized heme via the same set of enzymes that had been described for eukaryotes. However, the eukaryotic enzymes copro’gen oxidase and proto’gen oxidase, outlined above, utilize molecular oxygen in their reactions. This is clearly problematic for anaerobic/facultative bacteria which synthesize their own heme.^3,159^ A.F.M. Ehteshamuddin^160^ first approached this looking at anaerobic conversion of copro’gen to protoporphyrin by a species of *Pseudomonas*. The description of the activity in cell extracts was minimal, but did indicate that anaerobic conversion was possible by this organism. George Tait followed in 1969 with a study of this process in *R. sphaeroides*.^161^ During the 1960s, Tait, in collaboration with Neuberger and the support of Lascelles, had conducted investigations into the metabolism of *R. sphaeroides*, including heme and chlorophyll synthesis, during adaptation from aerobic, nonphotosynthetic growth to photosynthetic growth under anaerobic conditions (see ref 162). He discovered that a crude cell extract of anaerobically grown *R. sphaeroides* was able to convert copro’gen III to protoporphyrin IX in the absence of oxygen, but required Mg^2+^, ATP and l-methionine.^163,164^ Later he demonstrated in both *R. sphaeroides* and *Chromatium* that methionine could be replaced by S-adenosylmethionine (SAM), and the activity was inhibited by S-adenosylhomocysteine or S-adenosylethionine.^165^ Extracts from cells grown aerobically did not possess this anaerobic activity and required the presence of molecular oxygen to oxidize copro’gen. He noted that the aerobic conversion resembled that of the aerobic copro’gen oxidase activity previously described for liver mitochondria^126^ where molecular oxygen, but no cofactors, was required. Nicholas and Judith Jacobs’ group at Dartmouth Medical School examined the ability of a variety of bacteria to convert copro’gen to protoporphyrin in the presence and absence of oxygen. Interestingly, they found that the Gram-negative bacteria, *Pseudomonas fluorescans, P. denitrificans* and *E. coli* efficiently converted copro’gen into protoporphyrin, but the Gram-positive bacteria *Micrococcus lysodeikticus* and *Staphylococcus aureus* accumulated coproporphyrin and no protoporphyrin.^166,167^ Keithly and Nadler^168^ identified aerobic and anaerobic copro’gen oxidase activity in *Rhizobium japonicum*. These activities were similar to what Tait had identified in *R. sphaeroides.* It should be noted that this work was strictly biochemical, did not progress to the stage of enzyme isolation and characterization, and was done prior to the “genomic era” of biology.

Validation for the presence of an anaerobic copro’gen oxidase came after the advent of DNA technologies. This initially came from Thomas Elliott’s laboratory at the University of Alabama, Birmingham^169^ that carried out an insertional mutagenesis screen in *Salmonella typhimurium*. Their data demonstrated a double mutant of the genes named *hemF* and *hemN* resulted in heme auxotrophy. Mutation of *hemN* resulted in an inability to grow anaerobically but deletion of *hemF* had no impact on growth. They proposed that HemN was an anaerobic copro’gen oxidase and HemF was an oxygen-dependent copro’gen oxidase. The laboratories of Elliott^170^ with *S. typhimurium*, C.N. Hunter, at the University of Sheffield,^171^ with *R. sphaeroides*, and Dieter Jahn, at Philipps-Universität Marburg,^172^ with *E. coli* cloned and sequenced the genes and demonstrated that HemN was an anaerobic copro’gen oxidase. They confirmed the existence of separate aerobic (HemF) and anaerobic enzymes (HemN) in some bacteria to convert copro’gen III to proto’gen IX.

Coomber et al.^171^ also noted that *R. sphaeroides*, in which the HemN was knocked out, excreted large amounts of coproporphyrin into the medium when grown under low oxygen conditions. This is similar to an earlier observation by Lascelles, then at UCLA, that a mutant of *R. sphaeroides* (“2–33”) had an “enzymatic defect” in copro’gen oxidase and accumulated large amounts of copro’gen in the medium.^173^ An even earlier observation by Lascelles was that under iron limited conditions, *R. sphaeroides* excreted large amounts of coproporphyrin and that this was corrected by adding “catalytic” amounts of iron to the culture medium.^93^ The connection between these observations became clear when it was found that HemN possessed an essential [Fe–S] cluster^174^ and that this protein is member of the large “radical SAM” family. Many bacterial paralogs were initially misannotated as “HemN, probable oxygen-independent coproporphyrinogen oxidase”, so it appeared at that time that essentially all prokaryotes possessed HemN. This was demonstrated not to be accurate.^175,176^ Only Gram negative bacteria possess true oxygen-independent copro’gen oxidases. To untangle the annotation mess, new names were assigned to bacterial heme synthesis enzymes.^3^ The bacterial aerobic enzyme, now named copro’gen decarboxylase (CgdC), is highly homologous to the eukaryotic enzyme copro’gen oxidase, (CpoX) and distinct from the anaerobic enzyme, now named copro’gen dehydrogenase (CgdH). CgdH is unique to Gram negative prokaryotes (Figure 12).

**Figure 12:**
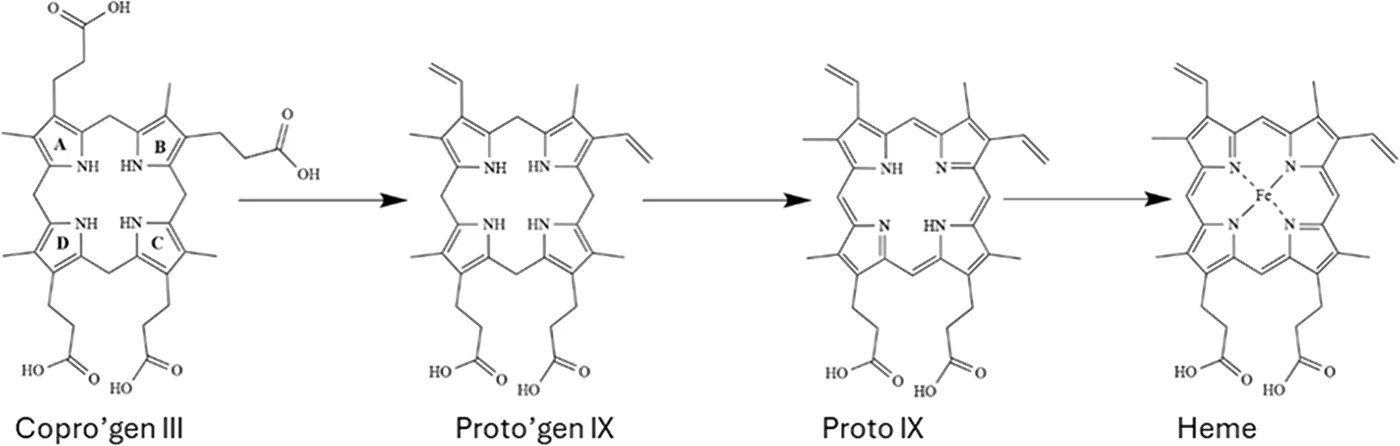
**P** rotoporphyrin **D** ependent **P** athway (PDP) from coproporphyrinogen III to protoheme IX. Conversion of copro’gen III to protogen IX is an oxidative decarboxylation of propionate side chains to vinyl groups on the A and B rings. The conversion of proto’gen IX to protoporphyrin IX is a six-electron oxidation. The enzymes catalyzing these two steps in all eukaryotes are oxygen-dependent, but some prokaryotes possess enzymes that catalyze the steps in the absence of molecular oxygen.

The other biosynthetic step in the pathway that requires oxygen in eukaryotes is proto’gen oxidase. Work from the Jacobs’ laboratory demonstrated that in *E. coli*, nitrate and fumarate could substitute for oxygen to support the oxidation of proto’gen to protoporphyrin and that menaquinone deficient mutants were unable to use fumarate as an electron acceptor.^177–179^ Thus, it appeared that this proto’gen oxidase was not truly an oxygen-dependent enzyme but simply linked to the electron transport chain and only required a suitable electron acceptor. Phage-mediated transduction of a heme deficient mutant of *E. coli* by Sǎsǎrman’s group at the University of Montreal assigned HemG as the proto’gen oxidase of *E. coli*.^180^ With the advent of gene sequencing it became clear that HemG has no resemblance to the eukaryotic proto’gen oxidase and is found only in enterics. This enzyme, now named proto’gen dehydrogenase (PgdH1), was independently characterized by the Dailey^181^ and Jahn^182^ research groups. It is an oxygen-independent, quinone-dependent flavodoxin that links to the respiratory chain. Data has also been presented demonstrating that in *Vibrio vulnificus* PgdH1 forms a complex with ferrochelatase (PpfC) to channel protoporphyrin between these two terminal pathway enzymes.^183^

A protein named HemY was initially characterized from the Gram positive *Bacillus subtilis* ^184,185^ as a homologue of the oxygen-dependent, eukaryotic flavoprotein proto’gen oxidase. Later HemY (now PgoX) was cloned, purified, and characterized from the Gram negative *Myxococcus xanthus*.^186^ However, this oxygen-requiring proto’gen oxidase (PgoX) is found in a limited number of Gram negative bacterial species and since it required molecular oxygen, it does not satisfy the need for an oxygen-independent proto’gen oxidase. The expansion of bacterial gene sequencing projects revealed that relatively few bacteria possessed either PgoX or PgdH1. So, most bacteria lacked a known functional enzyme to oxidize proto’gen. Resolution of this mystery came with independent studies and approaches by Kato et al.,^187^ and Boynton et al.^188^ They identified an enzyme (then named HemJ, now PgdH2) that catalyzes this step in the absence of oxygen. It is the most commonly found “proto’gen oxidase” enzyme in Gram negative bacteria and has no similarity in sequence to either PgoX or PgdH1. PgdH2 was shown to be a membrane-associated enzyme that is a *b*-type hemeprotein, functionally coupled with copro’gen oxidase, in *Synechocystis PCC 6803* (hereafter *Synechocystis*).^189^ The protein appears to be a stable dimer and is oxygen independent. An excellent review of the molecular phylogeny and evolutionary history of the three identified “proto’gen oxidases” found in prokaryotes is presented by Kobayashi et al.^190^

## 5 Alternate Pathway to Heme

Murphy and Siegel found that sulfite reductases in *E. coli* and *Desulfovibrio* species possess a unique heme moiety whose tetrapyrrole macrocycle is an isobacteriochlorin that they named siroheme^191,192^ (Figure 13). Whether siroheme was derived from protoheme, or originated from its own independent pathway was not determined until 1990. The first studies on the siroheme biosynthesis pathway began with the demonstration that uro’gen III was methylated to form dihydrosirohydrochlorin (now named precorrin-2) by an enzyme named CysG in *E. coli*.^193^ CysG was then shown by Scott’s research group to possess all enzyme activities required for synthesis of siroheme from uro’gen III.^194^ The gene *cysG*, that had been identified by Jeff Cole and suggested to be involved in siroheme synthesis,^195^ was then shown to encode the enzyme activities necessary to catalyze the methylation of uro’gen II to precorrin-2, the conversion of this to sirohydrochlorin by a dehydrogenase, and finally ferrous iron insertion to make siroheme.^196^ CysG contains two domains with the methyltransferase being located in the carboxy terminal segment (CysG^A^), and the dehydrogenase/ferrochelatase (CysG^B^) in the amino terminal domain. Interestingly CysG^B^ utilizes a single active site for both catalytic activities.^197,198^ This organization is not universal. For example, in *S. cerevisiae* two proteins carry out this pathway; Met8p possesses the dehydrogenase/ferrochelatase and Met1P catalyzes the methylation^199,200^ while in the firmicute bacterium, *Priestia* (previously *Bacillus*) *megaterium*, three separate proteins exist; SirA, SirB and SirC for the methylase, ferrochelatase, and dehydrogenase, respectively.^201^

**Figure 13:**
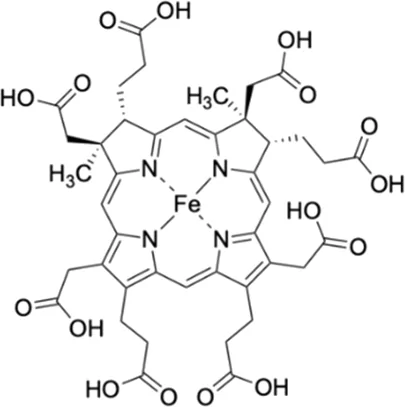
Chemical structure of siroheme.

It is of note that siroheme is considered to be the first “heme” molecule to have evolved. Details concerning siroheme biosynthesis are well reviewed and not repeated here.^3^ In 1993 Seiyo Sano’s laboratory provided data suggesting that the anaerobic bacterium *Desulfovibrio vulgaris* may utilize a different pathway to make protoheme than the “classical” pathway.^202^ They followed this observation with a more in-depth analysis of heme synthesis in *D. vulgaris*.^203^ Their significant contribution was demonstrating that protoheme formed in this organism possesses two methyl groups that do not originate from ALA but come from methylation of uro’gen III at the C-2 and C-7 positions with methionine to form precorrin-2. Rudolph Thauer’s group later described this for the methanogenic archaea *Methanoscarcina barkeri*.^204^

A major advance came from the genome-based examination of a number of *Archaea* that supported the presence of an alternate pathway to heme biosynthesis via extension of the siroheme synthetic pathway.^205^ Finally, it was demonstrated by Martin Warren’s group at the University of Kent^206^ that *Desulfovibrio* has the ability to convert siroheme to protoheme IX and heme d1 via the intermediates didecarboxysiroheme and coproheme (Figure 14). They named this the Alternative Heme Biosynthetic (AHB) pathway. Gunhild Layer’s group at Technische Universität Braunschweig then described this pathway in the methanogen *M. barkeri*.^207^ This pathway is oxygen independent, and its enzymes possess no similarity to any of the enzymes of the “classical” pathway. Four enzymes, AhbA-B, AhbC and AhbD are necessary. AhbA-B catalyze the conversion of siroheme into didecarboxysiroheme. AhbC catalyzes the oxygen independent, oxidative loss of two acetic acid side chains to form coproheme III. AhbD then catalyzes the oxygen independent, oxidative decarboxylation of two propionate side chains into vinyl groups thereby converting coproheme III into protoheme IX. AhbC and AhbD possess iron sulfur clusters and require S-adenosylmethionine for activity. The observation that coproheme III is synthesized by *D. vulgaris* explained the observation made in 2000 that these organisms have a bacterioferritin with bound coproheme III.^208^

**Figure 14:**
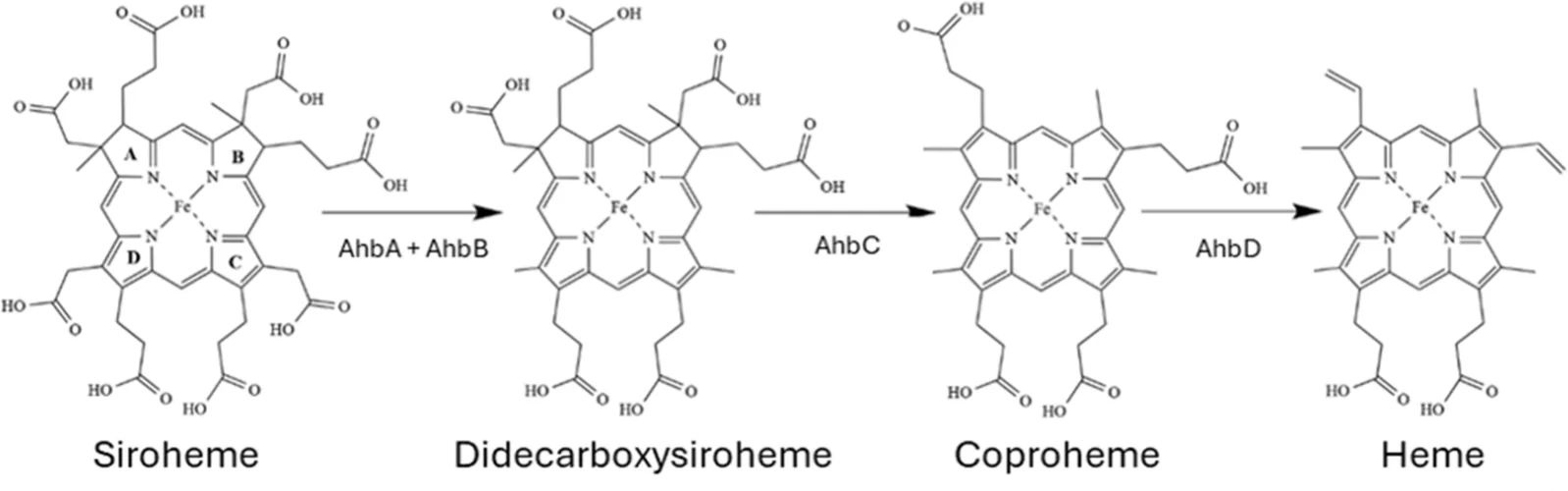
Steps in the **A** lternate **H** eme **B** iosynthetic (AHB) pathway from siroheme.

## 6 The Final(?) Missing Pieces

The genomes of Gram positive bacteria do not contain *hemF* (*cgdC*) but were predicted from genome annotations to encode HemN to catalyze conversion of copro’gen to protogen. However, “HemN” proteins from Gram positive bacteria lack copro’gen dehydrogenase activity; analysis of these proteins annotated to be “oxygen-independent copro’gen oxidase” revealed that they lacked the residues essential for coordinating the necessary [4Fe-4S] center.^209^ During the search for a functional copro’gen oxidase/dehydrogenase in Gram positive bacteria, a genome-based approach was taken Tamara Dailey, in the Department of Biochemistry and Molecular Biology at the University of Georgia, in collaboration with Svetlana Gerdes, then at FIG. This resulted in the identification of a protein they demonstrated was essential for heme synthesis in Gram positive bacteria.^209^ However, this protein, that they named HemQ, does not catalyze the conversion of copro’gen into proto’gen. What function HemG serves in heme biosynthesis was not resolved for another four years.

Determination of the role played by HemQ required a reexamination of data going back half a century. It had always been assumed that Gram positive organisms synthesized heme via the same pathway that had protoporphyrin as an intermediate. However, as early as 1948 it was known that the Gram positive bacterium, *Corynebacterium diphtheriae* produced and excreted large amounts of coproporphyrin, but no detectable protoporphyrin;^210^ Townsley and Neilands^211^ reported finding coproporphyrin III and coproheme III, but no protoporphyrin in the Gram positive bacterium *Microccus lysodeikticus*. Interestingly, even though coproheme was identified in cell extracts, they stated “. . .it is doubtful whether coproheme itself lies on the direct metabolic pathway of heme synthesis. . .”. Later, the Jacobs group found that while Gram negative bacteria produce protoporphyrin, they were able to identify coproporphyrin, but not protoporphyrin in Gram positive bacteria. Additionally, they reported that cell extracts of Gram negative bacteria converted copro’gen to proto’gen but extracts for Gram positive bacteria did not catalyze this reaction.^166,167^ Harms, Martinez and Griego^212^ determined that four species of *Bacillus* produced coproporphyrin but were unable to identify any protoporphyrin in these bacteria.

Finally, the Dailey laboratory^176^ demonstrated that in Gram positive bacteria the terminal portion of the heme synthesis pathway differs from the well-characterized classical pathway and that protoporphyrin is not an intermediate in the pathway to protoheme in these organisms. They demonstrated that Gram positive bacteria oxidize copro’gen to coproporphyrin, then insert iron to make coproheme, and finally decarboxylate coproheme to protoheme. This was named the coproporphyrin dependent (CPD) pathway^176,213^ (Figure 15) to distinguish it from the “classical”, or protoporphyrin dependent (PPD) pathway.

**Figure 15:**
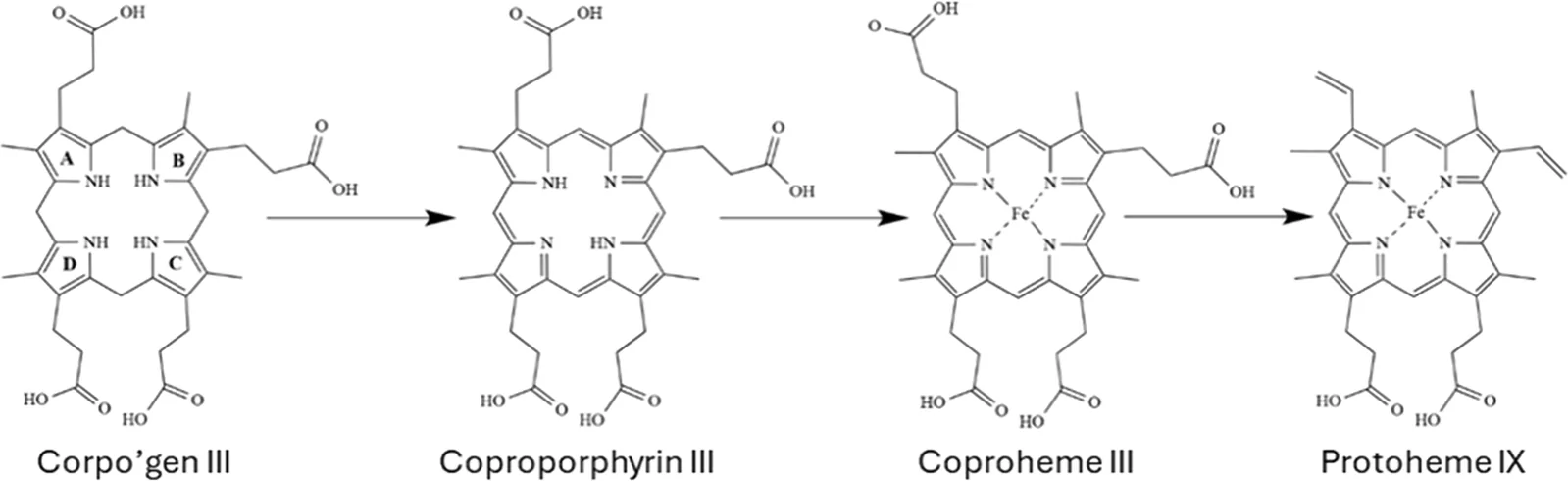
**C** oproporphyrin **D** ependent **P** athway from copro’gen to heme. The enzymatic steps for each segment are detailed in the text.

Two issues had masked the existence of the CPD pathway. The first was that the enzyme responsible for oxidizing copro’gen to coproporphyrin (CgoX) in the CPD pathway was originally characterized as HemY, a proto’gen oxidase. None of the characterized Gram negative bacterial or eukaryotic proto’gen oxidases oxidize copro’gen, but two research groups determined that the isolated HemY enzyme from *B. subtilis* would catalyze the oxidation of both copro’gen and proto’gen.^184,185^ Interestingly, Mats Hansson, while at Lund University, raised the possibility that HemY may oxidize copro’gen to coproporphyrin and that coproporphyrin was then converted to protoporphyrin in *B. subtilis*.^214^ The second issue was the enzyme responsible for inserting iron into coproporphyrin, coproporphyrin ferrochelatase (CpfC), was originally characterized as HemH (now PpfC), a protoporphyrin ferrochelatase.^215^ Thus, it appeared that Gram positive bacteria had all of the usual pathway enzymes except for coproporphyrinogen oxidase (HemF or HemN). It was only after the discovery of HemQ (now ChdC) and determination that it catalyzed the decarboxylation of coproheme III into protoheme IX, that things fell into place.^176^

However, CgoX was shown to require molecular oxygen and ChdC functions aerobically, so mechanisms must exist to catalyze these steps in facultative and anaerobic Gram positive bacteria. For an alternative to ChdC, Warren’s group had identified AhbD as an oxygen independent coproheme decarboxylase^206^ in the siroheme branch. Genomic analysis revealed that AhbD is present in anaerobic and facultative Gram positive bacteria including those that lack other AHB pathway enzymes.^175,176^ Additionally, it should be noted that ChdC was shown to utilize FMN in place of hydrogen peroxide,^176^ so an anaerobic conversion by ChdC *in vivo* may be possible. As for an oxygen independent copro’gen oxidase, Jahn’s group identified CgoN in *P. megaterium* as an oxygen independent copro’gen oxidase.^216^ Thus, it appears at present that the ability of all known heme synthesizing organisms to synthesize heme is covered by reported PPD, AHB and CPD pathway enzymes^3,175^ (Figure 16). Common to all heme synthesizing organisms is the core pathway of ALA to uro’gen III. Thus, the journey from the identification of glycine as a precursor to heme^44^ to the identification of the last heme biosynthetic pathway enzyme^216^ involved research groups from around the world and took almost eight decades (Table 1).

**Table 1:** Timeline of Heme Biosynthesis Pathway Discoveries (1840–2015)

**1840s: Birth of Porphyrin Research** - Chemists Berzelius, Scherer, and Mulder published techniques using concentrated sulfuric acid to remove iron from heme, then known as haematin.
**1867: Isolation of ″Cruetine″** - Thudichum produced and crystallized red, fluorescent ″cruetine″ (iron-free hematin) from blood.
**1871–1879: Coining the Name ″Porphyrin″** - Hoppe-Seyler coined the terms hematoporphyrin for blood-derived pigment and phylloporphyrin for metal-free chlorophyll.
**1874: Introduction of the Precursor Theory** - Baumstark proposed that porphyrins in clinical samples originated from heme precursor molecules rather than being breakdown products.
**Early 1900s: Establishment of Standard Nomenclature** - The names uroporphyrin, coproporphyrin, and protoporphyrin entered general usage following work by Willstätter, Küster, and Fischer.
**1912: Heme’s Skeletal Formula Proposed** - Küster proposed the correct skeletal formula for heme.
**1920s: Structural Determination of Protoporphyrin IX** - Fischer and his colleagues experimentally determined the tetrapyrrolic structure of protoporphyrin IX and the isomers of uroporphyrin and coproporphyrin.
**1930: Nobel Prize in Chemistry** - Fischer was awarded the Nobel Prize for his extensive work on the structure of porphyrins and heme.
**1937: Porphobilinogen Identified in Urine** - Waldenström first reported the presence of porphobilinogen (PBG) in the urine of patients with porphyria.
**1945–1946: Isotopic Tracer Breakthrough** - Shemin and Rittenberg used heavy nitrogen (15N) to prove that glycine is a direct nitrogen precursor to protoporphyrin.
**1948: Early Evidence of Pathway Diversity** - Gray and Holt reported that the bacterium *Corynebacterium diphtheriae* produced large amounts of coproporphyrin but no detectable protoporphyrin, an early hint of alternative pathways. The first successful in vitro synthesis of heme from glycine was achieved by the Shemin group.
**1950: Stoichiometry of Glycine Incorporation** - Muir and Neuberger determined that all four nitrogen atoms and eight specific carbons in the macrocycle originate from glycine.
**1952: Identification of the Succinate Contribution** - Shemin and Kumin demonstrated that ″activated″ succinate (later identified as succinyl CoA) provides the remaining carbon atoms for the porphyrin ring.
**1953: PBG and ALA Defined** - Cookson and Rimington defined the chemical structure of PBG as a substituted pyrrole. Shemin and Russell identified 5-aminolevulinic acid (ALA) as the first committed precursor of the pathway.
**1955: Discovery of Porphyrinogens** - Bogorad discovered that porphyrinogens, rather than porphyrins, are the true biosynthetic intermediates.
**1958: Characterization of Core Pathway Enzymes** - The enzymatic nature of ALA synthesis (ALA synthase) was characterized independently by groups led by Shemin and Neuberger. Bogorad identified the enzymes PBG deaminase and uroporphyrinogen isomerase. Uroporphyrinogen decarboxylase was also identified by Granick’s laboratory.
**1959–1960: Ferrochelatase and Mitochondrial Localization** - Labbe’s laboratory defined ferrochelatase as the enzyme responsible for iron insertion and located the process within the mitochondria.
**1961: Coproporphyrinogen Oxidase** - Sano and Granick demonstrated the conversion of coproporphyrinogen III to protoporphyrinogen IX by coproporphyrinogen oxidase.
**1969–1970: Identification of Anaerobic Adaptations** - Tait and the Jacobs group identified oxygen-independent pathway steps in bacteria.
**1972: Mitochondrial Ferric Reductase** - Jones demonstrated that mitochondria reduce ferric iron to ferrous iron for utilization by ferrochelatase.
**1973: Discovery of the C5 Pathway** - Beale provided the first clear evidence for the C5 (glutamate-based) pathway to ALA in plants.
**1975: Identification of Protoporphyrinogen Oxidase** - Poulson and Polglase identified and purified protoporphyrinogen oxidase.
**1990: Siroheme Synthesis Elucidated** - The *cysG* gene in *E. coli* was shown to encode the activities necessary to synthesize siroheme from uroporphyrinogen III.
**2011: Formal Discovery of the AHB Pathway** - Warren’s group demonstrated that *Desulfovibrio* has the ability to convert siroheme to protoheme IX via didecarboxysiroheme and coproheme.
**2015: Formal Discovery of the CPD Pathway** - The Dailey laboratory demonstrated that in Gram positive bacteria, the terminal portion of the pathway differs significantly, with coproheme—not protoporphyrin—serving as the key intermediate.

**Figure 16:**
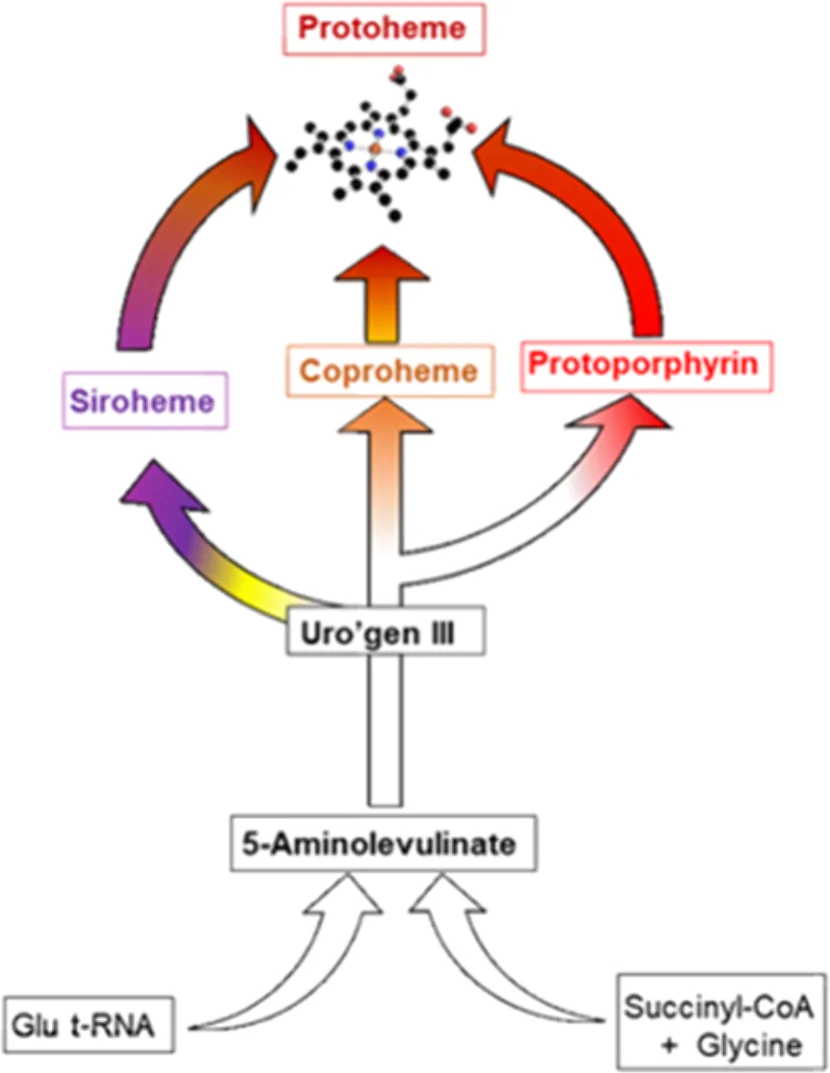
Three pathways to synthesize heme.

## 7 Characterization of Pathway Enzymes

As the steps in the pathway were identified, the next goal was to characterize the individual enzymes. Much of the published research during the 1970s and 1980s focused on purifying enzymes out of primary tissue/cell sources and kinetically characterizing enzymes responsible for each step. For modern scientists obtaining a purified protein is relatively easily accomplished by cloning and expressing large quantities of recombinant, affinity-tagged protein that is then quickly purified with a single column. Purification in the 1970s had no access to such technology and relied upon breaking down whole organs, tissues, or cells before disruption, and fractionation. Indeed, a protein lab at that time would have an assortment of centrifuges, and cold boxes/rooms filled with meter-long columns connected to 100-tube fraction collectors. It was a different world where purifying a few milligrams of protein could take several days. Such long times frequently led to undesired and unrecognized proteolysis and loss of cofactors. Thus, it is understandable that different research groups sometimes presented significantly different results for the same enzyme. Availability of recombinant protein produced in bacteria, yeast or baculovirus systems was a major step forward, but comes with the caveat that “normal” post-translational protein modifications, such as phosphorylation, glutathionlyation, acetylation, carbohydrate attachment, etc., will not in occur in the expression system unless specific efforts are made to support these modifications.^17^ Below I review the various advances, but do not focus on describing enzyme structures and mechanisms since that information is beyond the scope of the present work. A number of excellent reviews are available as are primary publications that are focused on individual enzymes.^16,217^

The desire to characterize each enzyme in absolute isolation *in vitro*, while worthy of the moniker “reductionist biochemistry”, is understandable and provides great scientific value. However, *in vivo* pathway enzymes exist in a gel-like environment, in proximity to numerous other proteins. Once purified each enzyme was subjected to kinetic analysis to determine affinity for substrates, inhibitors and products, along with catalytic efficiencies. In general, assay conditions were optimized for individual enzymes and sometimes included using nonphysiological substrates. This approach, unfortunately, ignores the *in situ* reality in which these enzymes function where protein–protein interactions and *de facto* substrate channeling exist. But that is a discussion for a metabolism review rather than an historical review. In reviewing this part of history, I will concentrate on each enzyme individually rather than follow a time-line based presentation.

### 7.1 ALA Synthesis

As detailed above, ALA is the first committed intermediate in the synthesis of all tetrapyrrole compounds. While ALAS was one of the first enzymes identified for heme biosynthesis, it was undoubtedly the last pathway enzyme to have evolved. The earliest heme synthesis pathways formed ALA via the C-5 pathway with ALAS first appearing in the alpha-proteobacteria. However, there may be an evolutionary link between ALAS and the C-5 pathway. The structural similarity between ALAS and GSA aminomutase, both pyridoxal phosphate utilizing enzymes, led to the postulation that ALAS evolved from GSA aminotransferase.^218^

### 7.2 ALA Synthase

Since ALA synthase was considered to be the rate-limiting step in heme synthesis, many groups using a variety of organisms jumped into the ALA synthase purification fray. ALAS activity was first reported in cell extracts in 1958 and there was general agreement that the enzyme is a homodimer with pyridoxal phosphate as a noncovalently bound cofactor.^87–89,219^ The first significant effort to purify the enzyme was by Burnham and Lascelles^220^ as well as Warnick and Burnham^221^ utilizing *R. sphaeroides.* They reported a purification of 1,300-fold, a molecular weight of 57,000 Da, 50% inhibition of activity by 5 μM hemin and K_m_s for glycine and succinyl-CoA of 10 mM and 25 μM, respectively. Davies and Neuberger^222^ later reported the purification to apparent homogeneity of AlaS from *R. sphaeroides.* They reported a subunit molecular weight of 49,000 Da.

Several investigators reported the presence of an oxygen-sensitive endogenous activator of the *R. sphaeroides* AlaS that converted the enzyme from a low activity form into a high activity form.^223–225^ This compound was identified as an endogenous, oxygen-sensitive trisulfide.^226^ No other ALA synthase enzymes have been reported to be activated in a similar fashion, so this oxygen-sensitive modulation of AlaS activity in *R. sphaeroides* probably evolved to satisfy the physiology of the facultative photosynthetic bacteria. The gene encoding AlaS from *R. sphaeroides* was cloned by Kaplan’s group,^227^ and the protein was expressed, purified and characterized a decade later by Warren’s group.^228^ That AlaS has a subunit molecular weight of 44,558 Da, purifies as a dimer with pyridoxal phosphate and has K_m_s of 1.8 mM and 17 μM for glycine and succinyl CoA, respectively. The gene encoding AlaS from the related organism *Rhodobacter capsulatus* was cloned in 1988 ^229^ and the crystal structure of the purified expressed protein was determined.^230^ The *R. capsulatus* protein is highly similar to that of *R. sphaeroides*.^231^ The only other bacterial AlaS studied in any detail is that of *Paracoccus* (originally *“Micrococcus*”) *denitrificans*. Tait^232^ partially purified and characterized that AlaS reporting a molecular weight of 68,000 Da, and K_M_s for glycine and succinyl CoA of 12 mM and 10 μM, respectively. Hemin at 2 μM inhibited the enzyme activity by 50%. The cellular level of activity of the enzyme varies considerably depending upon growth conditions. *P. denitrificans*, like *R. sphaeroides*, is a member of the alpha-proteobacteria, the only group of bacteria that possess the enzyme AlaS. It is an interesting bacterium since, because of its metabolic machinery, it has been suggested to be the ancient precursor to eukaryotic mitochondria.^233^

Goro Kikuchi, who was introduced to the heme synthesis field while in Shemin’s laboratory on a leave from Tohoku University School of Medicine, Sendai, demonstrated that ALAS was found in both liver mitochondria and cytosol of 2-allylisopropylacetamide (AIA)-treated rats.^234^ Scholnick, Hammaker and Marver^235,236^ reported a partial purification and kinetic characterization of ALAS from rat liver, and Aoki et al.,^237^ presented a purification from rabbit reticulocytes. However, the first to achieve significant purification of ALAS from an animal source were Whiting and Granick.^238^ They utilized liver from chicken embryos that had been pretreated with AIA and 1,4-dihydro-3,5-dicarbethoxycollidine (DDC) to induce ALAS levels *in situ*. ALAS was released from mitochondria by sonication prior to numerous purification steps. The purified ALAS was found to have a single band on SDS electrophoretic gels and a subunit molecular weight of 49,000 Da. The fact that this was a proteolytic product and not intact ALAS came later from Whiting, then at University of Adelaide, Australia. Using isolated chick embryo liver polysomes, he demonstrated that *in vitro* synthesized ALAS had a molecular weight of 70,000 Da.^238^ He proposed that this was processed after translocation into the mitochondrion to a 51,000 Da mature protein. Soon after, Diana Beattie’s group at Mount Sinai School of Medicine in New York reported the purification of ALAS from rat liver mitochondria.^239^ Their prep also appeared homogeneous on an SDS gel and had an estimated subunit molecular weight of 58,000 Da. Kikuchi’s lab reported the purification of ALAS from rat liver cytosolic fraction.^240^ Their ALAS was reported to be a dimer of two identical subunits of molecular weight of 51,000 Da. As had been mentioned in many previous studies, one difficulty in purifying ALAS was that it was always found as a “complex” with other “associating proteins”. Nakakuki et al. circumvented this problem by treatment of their extracts with the protease, papain. Unfortunately, all of these ALAS enzyme preps were proteolytic fragments.

Working with the chick embryo system, Ades and Harpe at Wake Forest University followed up on the earlier work by Whiting,^241^ and Brooker, May and Elliott^242^ using *in vitro* translation to synthesize ALAS. They presented immunological evidence for the presence of a 75,000 Da precursor form of ALAS and a 65,000 Da mitochondrial ALAS.^243^ They later purified ALAS and reported a molecular weight of 63,000 Da, although the specific activity of their ALAS was significantly lower than that reported previously by others.^244^ The May and Elliott group at the University of Adelaide, Australia, reexamined both chick embryo liver ALAS and rat liver ALAS. For chick hepatic ALAS they found a subunit molecular weight of 68,000 Da^245^ which contrasted to the 49,000 Da that was reported by Whiting and Granick. Proteolytic treatment of the 68,000 Da form of the enzyme yielded a 50,000 Da protein that retained enzyme activity. For the rat liver ALAS^246^ they isolated a protein with a subunit molecular of 70,000 Da that was diminished to 56,000 Da following papain treatment as had been done by Nakakuki et al.^240^ Kinetic characterization of all ALAS enzymes, regardless of source, gave similar results. The K_m_s for the substrates are generally in the range of 2.5 to 10 mM for glycine and 10 to 25 μM for succinyl CoA.

As noted above, Kikuchi’s group had reported that ALAS was present in both the cytosol and mitochondria in rat liver.^234^ They noted that the cytosolic form had a half-life of 20 min, shorter than the 68 min for the mitochondrial form. They hypothesized that ALAS was synthesized in the cytosol before being transferred into the mitochondrion. Later this group demonstrated that exogeneously administered hemin inhibited the translocation of the cytosolic liver ALAS into the mitochondrion.^247^ These studies were expanded by Ades and Hape^243^ to include the ALAS of embryonic chick liver. They used an immunological approach to monitor cytosolic vs mitochondrial “location” by the decrease in ALAS size from 74,000 Da to 68,000 Da after translocation and proteolytic processing. This observation was enhanced by Srivastava et al.^248^ who isolated mitochondria away from the cytosolic fraction prior to examining the relative amounts of radiolabeled ALAS by immunological detection. These were noteworthy observations since the first cDNA sequence for ALAS that included the mitochondrial targeting sequence was not published until 1985 by Borthwick et al.^249^ and the first recombinant expression and purification of a mammalian ALAS was not until 1993.^250^

A hotly debated ALAS-related controversy was settled in 1989. For over a decade there had been publications and symposia discussions about whether there were two forms of ALAS in higher animals. Indeed, there were strong arguments posited by many that there was only a single genomic ALAS. David Bishop, while a graduate student in W.A. Wood’s laboratory at Michigan State University,^251^ had presented data in support of distinct erythroid and hepatic forms of ALAS, but their conclusions were based upon biochemical and kinetic differences that they observed with ALAS fractions isolated from erythroid vs hepatic sources. It was not clear if this represented two genetically distinct forms of the enzyme, or if a single gene product may have been post-translationally modified. The issue was finally and definitively settled by the publication of Riddle, Yamamoto and Engel.^252^ They demonstrated that avians possess two genetically distinct forms of ALAS; one found in all nonerythroid cells (ALASN, now ALAS1) and another found only in developing erythroid cells (ALASE, now ALAS2). These two ALASs are encoded by two distinct genes, although they have very similar amino acid sequences and appear to have originated from an ancient gene duplication event. In humans, ALAS1 is encoded on chromosome 3p21 ^253^ and ALAS2 on the X chromosome.^254,255^ Of note, while ALAS2 is sex-linked in mammals, this is not true for all animals. For example, in chickens, ferrochelatase, rather than ALAS2, is sex linked on the Z chromosome (male: ZZ, female: ZW).^256^ Biochemical support for an essential role of ALAS2 in erythroid cells first came from studies in murine erythroleukemia cells where it was demonstrated that erythroid differentiation only occurred with functional ALAS2 expression and that ALAS1 expression diminished early during erythroid differentiation.^257^ Later it was shown in cultured mouse embryonic stem cells that induction of a functional ALAS2 was essential for the formation of hemoglobin in erythroid islands.^258,259^ More recently it has been reported that ALAS2 expression is not restricted to just developing erythroid cells, but is also the form of ALAS expressed and utilized in some mammalian brain cells^260,261^ and that the frequently utilized chemotherapeutic drug doxorubicin causes significant down-regulation of ALAS2 in hippocampal neurons.^262^

The evolution of two distinct forms of ALA synthase appears to have occurred in the *Mollusca*. The existence of “red blood” occurs in a limited number of species of invertebrates: *Phoronida, Annelida, Nemertina, Echiuroidea, Mollusca*, and *Echinodermata*.^263^ With few exceptions, such as the annelid *Capitella*,^264^ the red blood is attributable to circulating macromolecular complexes of hemoglobin and not hemoglobin-containing cells. Presently available data reveal that it is in the blood clams *Tegillarca granosa* and *Scapharca broughtonii* where one first finds two genetically distinct ALAS synthases with one, ALASII, specific for heme synthesis in hemolymph cells. Other clams that do not possess “red cells” lack two ALAS genes.^265^

With the availability of ALAS2 sequence, it was demonstrated that the cytosolic precursor form of ALAS2 possesses an amino-terminal mitochondrial targeting sequence containing two heme-binding motifs that inhibit translocation of the precursor ALAS2 into mitochondria *in vitro*.^266^ Similar findings were later reported for AlaS translocation in the yeast, *Kluyveromyces lactis*.^267^ Mammalian ALAS1 and ALAS2 have a third heme binding motif near the amino-terminal end of the mature, proteolytically processed protein. Using green fluorescent protein-tagged ALAS1, Dailey’s laboratory demonstrated that heme inhibition of ALAS1 translocation in cultured murine erythroleukemia cells is modulated by all three heme binding motifs (two in the mitochondrial targeting sequence and one at the amino terminus of the mature protein).^268^ Munakata et al.^269^ examined the impact of heme on the translocation of rat ALAS1 and ALAS2 into mitochondria of cultured quail QT6 fibroblasts. They found that only the first and third heme binding motifs modulated heme-sensitive import of ALAS1 into mitochondria, and that ALAS2 translocation was not inhibited by hemin in their quail fibroblast system. Of the four mitochondrially located heme synthesis enzymes (CPOX, PPOX, FECH and ALAS), only ALAS translocation has been found to be sensitive to heme in intact cells.^268^

Heme inhibition of purified ALAS has been examined by a number of laboratories. The reported concentrations of hemin required for approximately 50% inhibition of ALAS activity range from a low of 3 μM hemin for purified enzyme from *R. sphaeroides* ^270^ to greater than 1 mM for ALAS from chick liver.^271^ Studies by Wolfson, Bartezak and Bloomer^272^ at Yale University School of Medicine, utilized intact rat liver mitochondria and measured the *in situ* activity of ALAS in the presence or absence of intramitochondrial heme synthase (ferrochelatase). Their data indicated that even when ferrochelatase was synthesizing above normal amounts of intramitochondrial heme from protoporphyrin and ferrous iron, ALAS activity was not diminished, suggesting that *in vivo* ALAS activity is not inhibited by heme. However, the demonstration that ALAS and FECH are part of a mitochondrial heme metabolon complex raises the possibility that because of proximity, *in situ* synthesized heme may have an impact on spatially close ALAS.^273–275^ Recently Breann Brown’s research group at Vanderbilt^276^ demonstrated that recombinantly expressed, mature human ALAS2 is inhibited with an IC_50_ of 20 μM heme and that the protein has multiple heme binding sites. They propose that heme binding to ALAS2 may alter the tertiary structure of ALAS2 such that its interactions with other heme metabolon proteins may be affected.

While the assertion that ALAS is the rate limiting step in heme synthesis is frequently stated, available data do not support this for all organisms or tissues. Woods and Dixon^277–279^ reported that in prenatal tissues of rats, rabbits and guinea pigs, ALAS activity is high but decreases upon birth. This work was extended to show that unlike in adult rat liver, ALAS activity is not limiting for heme production in fetal rat liver.^277^ In yeast and fungi, data are consistent with PbgS, not AlaS, being the rate limiting step.^280,281^ In chicken eggshell gland, ALAS1, along with ABCG2, are expressed at high levels and CPOX and FECH may be rate limiting for protoporphyrin and heme synthesis, respectively.^256,282^ Likewise, in certain clams that produce porphyrins for shell coloration, PpoX and FecH are rate limiting.^283^

To date, three crystal structures of ALAS are available: AlaS from the bacterium *R. capsulatus*,^230^ AlaS from the yeast *S. cerevisiae*,^284,285^ and ALAS2 from human.^286^ All have highly homologous catalytic cores, but the eukaryotic ALAS are initially synthesized with an amino-terminal mitochondrial targeting sequence that is removed upon translocation into the mitochondrion, and a carboxyl-terminal extension that varies between ALAS1 and ALAS2. The *R. capsulatus* protein lacks both the amino terminus, and the carboxy terminus found on the eukaryotic enzymes. The role(s) of the carboxyl terminal sequence of eukaryotic ALAS enzymes studied to date is not uniform. For the human ALAS2, it is involved in modulation of enzyme activity and known to interact with SUCLA2.^276,286–289^ The role it may play in ALAS1 or other nonerythroid metazoan ALASs is currently unidentified.

ALAS gene and enzyme regulation are beyond the scope of the current review. Considerable studies on ALAS regulation have been carried out by a number of research groups. Two useful reviews are by Sadlon et al.^290^ as well as Medlock and Dailey.^17^ Likewise, review of enzyme mechanisms and structure exceeds the current goals, but can be found in Astner et al.,^230^ Layer et al.,^16^ Stojanovski et al.,^291^ and Bailey et al.^286^ Of interest is a publication presenting evidence that cytosolic apo-ALAS1 may play a noncanonical role as a heme-independent inhibitor of small RNA-mediated silencing.^292^

### 7.3 C5 Path to ALA

The history of research for the C5 pathway is briefer than for ALAS due to its more recent discovery. However, the pathway is more abundant in Nature being found in the great majority of prokaryotes and all plants, and it has become an increasingly frequent target of research. Because of the necessity to produce protoporphyrin for both heme, chlorophyll and a variety of bilins, its regulatory mechanisms have proven to be more diverse. As outlined above, the two key discoveries were the determination that glutamate, and not glycine, was the precursor to porphyrin,^136–138,293^ and that glutamyl-tRNA was essential.^153,154,294^

The two enzymes necessary for ALA synthesis are glutamyl-tRNA reductase (GluTR), and GSA aminomutase (aminotransferase) (GsaM). The genes for both of these proteins have been cloned and the proteins isolated from a number of photosynthetic and nonphotosynthetic organisms (see refs 217 and 295 ). The crystal structure of GluTR from *Methanopyrus kandleri* has been determined and that protein has a distinctive “V” shape.^296^ GsaM has been crystallized from the nonphotosynthetic organisms *B. subtilis* ^297^ and *P. aeruginosa* ^298^ as well as the photosynthetic *Synechococcus* ^299^ and the plant *Arabidopsis thaliana*.^300^ The intermediate compound, glutamate-1-semialdehyde (GSA), is unstable, so it is not surprising that GluTR and GsaM interact to facilitate transfer between the enzymes^295,301,302^

Regulation of heme synthesis in C5 pathway organisms examined to date indicate that production of ALA is the primary rate limiting step in both photosynthetic^303^ and nonphotosynthetic^304^ organisms.

### 7.4 PBG Synthesis

As mentioned above, PBG synthase (PBGS, ALA dehydrase/dehydratase) activity and partial purification was first reported by Neuberger’s group^76,77^ from ox liver, Granick from chicken erythrocytes^78^ and Schmid and Shemin^305^ from duck erythrocytes. The first substantial purification of PBGS was from mice by D. L Coleman,^306^ and thereafter a significant number of groups reported purification and characterization of the enzyme. Among these are PBG synthase from yeast,^307^ *R. sphaeroides*,^308–310^ Tobacco leaves,^311^ *Aquaspirillum* (previously *Spirillum*) *itersonii*,^312^ beef liver,^313,314^ human erythrocytes,^315–317^ *Neurospora crassa*,^318^ dog liver,^319^ spinach,^320^ and *E. coli*.^321^ In all instances the enzyme is soluble and in all metazoans it is located in the cytosol.

By 1990 the cDNAs for PBG synthase had been isolated for human,^322^ rat,^323^ yeast^324^ and *E. coli*.^325^ In humans, the single gene for PBGS is assigned to chromosome 9q34.^326,327^ Aternative splice variants exist for housekeeping vs erythroid-specific expression of the gene,^328^ but the alternate splice sites are in the noncoding region, so the synthesized protein is identical in both erythroid and nonerythroid cells. Interestingly some domestic mice have been reported to have duplication and triplication of the PBGS gene that results in elevated levels of PBGS.^329^ This elevation was suggested to provide the mice with increased resistance to heavy metal toxicity. This may be a reasonable assumption given that it has long been known that lead inhibits PBGS and that measurement of PBGS activity and/or accumulation of ALA can serve as indicators of lead poisoning in humans.^330–332^ Indeed, a significant portion of the published literature on PBGS is related to lead toxicity.

The enzyme from all sources is multimeric. Currently available crystal structures of PBGS reveal a homo-octomeric quaternary structure (see refs 333–336 ). The enzyme may be dissociated into individual subunits of approximate molecular weight of 35,000 Da, and reassociated *in vitro*.^314,337^ Eileen Jaffe, at Fox Chase Cancer Center, found that the enzyme may exist as both a “high activity” homo-octomer and a lower activity homohexamer.^333,336,338^ The shift between the octomeric and hexameric forms, referred to as morpheens, is postulated to occur naturally in cells. However, protein variants that are found in “ALAD porphyria” (also called Doss porphyria or plumbo porphyria) are suggested to shift the equilibrium toward the low activity hexameric form.

Numerous studies have shown that the enzyme possesses essential bound Mg^2+^ or Zn^2+^ (reviewed by^333,339^ ). However, more recently it was shown that mammalian PBGS has a [4Fe-4S] cluster and that the presence of this cluster favors the high activity octomeric structure of PBGS.^340^ The suggestion made for why the cluster was not recognized sooner is that the iron sulfur clusters may not be formed in the recombinantly synthesized proteins and are unstable to the aerobic conditions existing in the purification schemes. In the absence of the cluster, Zn or Mg may become incorporated instead. The substitution of Zn for a cluster is well established for a number of recombinantly produced proteins.^341^ Whether PBGS exists *in situ* as a metalloenzyme with bound Zn or Mg, as a [4Fe-4S] cluster-containing protein, or a combination of both is not known. This is clearly a question in need of additional experimental data. Of interest is that PBGS was suggested to serve moonlighting functions as a proteosome-interacting protein.^342,343^ It was reported that PBGS may interact with the proteosome to modulate its activity in a dose dependent manner. At the present time, these observations have not promoted additional studies.

Detailed discussion of PBGS structure and catalysis is outside of the realm of the current review, but has been covered in excellent detail by Schubert, Erskine and Cooper,^339^ and Jaffe.^333^ The reaction catalyzed is a Knorr-type condensation as mentioned above. Each subunit possesses a complete active site to which two molecules of ALA bind. The first ALA to bind contributes what will become the propionate side chain of the pyrrole and the second ALA to bind contributes the acetate side chain. These sites are referred to as the P and A substrate binding sites, respectively. Crystal structures exist of a number of PBGS molecules with bound substrate and/or inhibitor which support the proposed catalytic mechanism and identify essential amino acid side chains that participate in the reaction. Both levulinic acid and succinylactone inhibit the enzyme^344^ and, thereby, heme synthesis *in situ*. These compounds have been employed frequently to inhibit heme biosynthesis in cell culture.

### 7.5 Synthesis of Uroporphyrinogen III

The polymerization of four molecules of PBG to form the cyclic tetrapyrrole uroporphyrinogen III is, perhaps, the most widely investigated step in heme biosynthesis. It is of interest not because of the polymerization reaction *per se*, but because to produce the III isomer requires that the “D” ring be inverted relative to the other three pyrrole rings. The early history of how the two enzymes responsible for this reaction, PbgD and UroS, were identified is covered above. It was shown that PbgD could catalyze the formation of a linear tetrapyrrole that will spontaneously cyclize to form the I isomer. It is only in the presence of UroS that the III isomer was formed. But neither the chemistry of the process nor the possible presence of any essential cofactor was known. How these details were eventually experimentally determined is a long story that is presented in excellent fashion by P. M. Jordan^107^ and F.J. Leeper.^106^ It is a complex and frequently confusing history with numerous flawed proposed models.

As covered above, early purification efforts clearly revealed that the enzyme acting alone was responsible for the synthesis of what Bogorad called a polypyrromethane. PBG deaminase was purified from a variety of sources including *R. sphaeroides*,^345,346^ spinach,^347^ human erythrocytes,^348^ *Chlorella*,^349^ *Euglena gracilis*,^350^ rat liver,^351^ and *E. coli*.^352,353^ In all cases the enzyme is located in the cytosol as a soluble monomer with reported molecular weights ranging from approximately 34,000 Da to 44,000 Da, and has a K_M_ for PBG in the low micromolar range. The gene for human PBGD is located on chromosome 11q24.1...q24.2.^354–356^ While there is only a single copy of the gene, alternate mRNA splice variants exist to yield housekeeping and erythroid forms with the erythroid form having an additional 17 amino acids at the amino terminus.^357,358^ This extension does not seem to have an impact on catalysis, but its role *in situ* has not been examined.

Much of what we know about the catalytic cycle comes from Ian Scott’s laboratory at Texas A&M, Peter Jordan’s group at University of Southhampton, and Alan Battersby’s group at Cambridge. In a series of experiments that utilized ^13^C NMR spectroscopy of pyrrole compounds produced by the enzyme, along with enzyme assays, it was clearly shown that the product of the reaction was the linear tetrapyrrole, hydroxymethylbilane (HMB). With the identification of HMB as the product of the enzyme catalyzed reaction, PBG deaminase was renamed HMB synthase (HmbS). HMB rapidly cyclizes into uro’gen I in the absence of UroS.^106,107,359^ The fact that the enzyme assembles four PBG molecules head to tail starting with the “A” ring first then progressing to B, then C and finally D, was demonstrated by Jordan and Seehra^360^ employing ^14^C labeled PBG, and by Battersby et al.^361^ who utilized ^13^C labeled PBG for NMR studies. Independently, at Mount Sinai, Anderson and Desnick^348^ demonstrated that HMBS (called uro’gen I synthase in that publication) purified from human erythrocytes could be isolated with one, two, three or four covalently bound PBG molecules, i.e. ES, ES_2_, ES_3_, ES_4_. Similar results were reported by Jordan’s group for the enzyme from *R. sphaeroides* ^362^ and *E. coli*.^363^

One critical piece of information was to identify the site of attachment of the first pyrrole (that would become the A ring) on the enzyme. This story was only resolved after the gene for HmbS was cloned, and the protein expressed recombinantly and purified in relatively large quantities.^364^ Through a series of elegant experiments by the Jordan laboratory, it was shown that the enzyme is synthesized as an apoprotein that lacks any cofactors. The fact that mutants of *E. coli* which were unable to synthesize ALA or PBG lacked HmbS activity, even though they synthesized the HmbS protein,^365^ presented a clue. It was demonstrated independently by Battersby’s group^366^ and Jordan and Warren^363,367,368^ that HmbS possesses a covalently bound dipyrromethene cofactor that is assembled during the first two turnovers of the apoprotein. Thus, apo-HmbS covalently binds the first molecule of PBG via a covalent linkage with a conserved cysteine residue,^369,370^ and then adds an additional five PBGs to form a covalently assembled linear hexapyrrole. The terminal tetrapyrrole is split off, leaving behind the dipyrromethene cofactor to serve as the enzyme-bound cofactor for subsequent catalytic cycles.

An understanding of how HMB synthase may function has resulted from the examination of two dozen crystal structures of the holoenzyme from a variety of sources. The first of these was the 1.76 Å crystal structure of *E. coli* HmbS.^371^ An overview of structures available prior to 2021 is presented in an excellent short review by J. R. Helliwell.^372^ Additional information on enzyme structure/function can be found in publications focused on crystal structures of human HMBS with and without inhibitors bound,^373^ with a bound ES3 intermediate^374^ and in a molecular dynamics examination of the human enzyme.^375^

The formation of uro’gen III from HMB is carried out by the enzyme now named uroporphyrinogen III synthase (UroS). As detailed above, this enzyme was first identified by Bogorad and named uro’gen III isomerase.^114^ It was known that in the presence of both HmbS (then named PbgD) and UroS, uro’gen III was formed from PBG, but UroS alone was unable to convert uro’gen I into uro’gen III. It was proposed by Frydman at the Universidad de Buenos Aires that the two proteins physically interact since HMB is reactive and will chemically cyclize to uro’gen I within minutes. One proposal was that UroS acts as a “specifier protein” causing HmbS to produce uro’gen III (see refs 105 and 376 ) However, data from a number of laboratories were not consistent with this proposal. After the identification of HMB as the product of the enzyme HmbS, it was clear that the substrate for URO synthase was HMB and not uro’gen I. Thus, URO synthase must catalyze the ring closure while flipping the D ring to form the III isomer. The mechanism whereby this is accomplished was first suggested by J.H. Mathewson and A.H. Corwin in 1961 to involve a spiro mechanism,^104^ but data to support that hypothesis were not available until Battersby’s group conducted extensive ^13^C based experiments that clearly supported a spiro mechanism to flip the D ring (see ref 377)­(Figure 17). A thorough discussion of this work is outside the scope of this review, but it well is reviewed by Schubert, Erskin and Cooper.^339^

**Figure 17:**
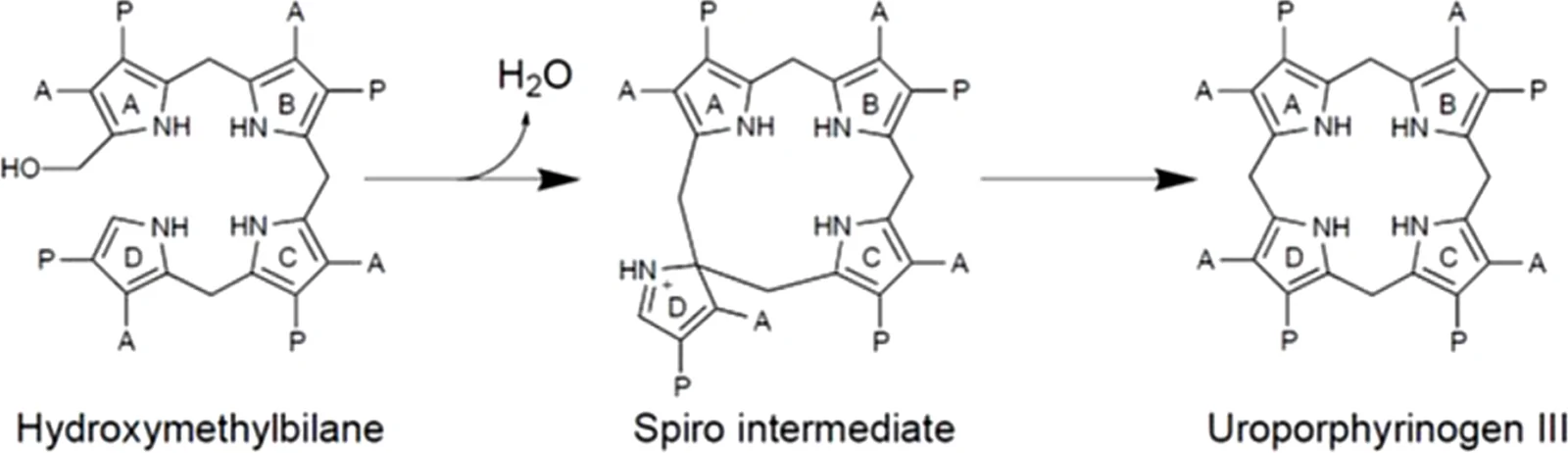
Spiro mechanism whereby the D ring of the linear tetrapyrrole is flipped during ring closure to form the III isomer of uroporphyrinogen.

URO synthase has been purified from cow liver,^378^ soybean,^379^ rat liver,^380^ *Euglena gracilis*,^381^ human erythrocytes,^382^ *E. coli*,^383^ *B. subtilis*,^384^ mouse,^385^ *A. thaliana*,^386^ and *Thermus thermophilus*.^387^ It is a cytosol-located, soluble monomer of approximate molecular weight of 30,000 Da, with no attached cofactor. Interestingly, while the primary sequences of all other heme biosynthesis enzymes are highly conserved, this is not true for URO synthase. As noted by others,^339,386^ there is only 14% identity between UROS of human and UroS of *T. thermophilus*.^387^ Indeed, there are only seven invariant residues among all enzymes identified to date. Because of this, there are heme synthesizing organisms for which we currently lack an annotated UroS. There exist a limited number of crystal structures,^387,388^ but structures of the *T. thermophilus* with and without bound product give hints as to its functioning.^387^

The human UROS was first cloned in 1988 ^389^ and its chromosomal location assigned as 10q25.5-q26.3 in 1991.^390^ As with all mammalian heme biosynthetic pathway enzymes, the UROS gene has alternative promoters for house-keeping vs erythroid-specific expression.^391^ Similar to PBGS and HMBS, the formation of UROS mRNA is subject to alternate splicing for erythroid vs nonerythroid synthesis. However, the expressed UROS protein is identical between house-keeping and erythroid-specific forms since the alternate splice site junctions involve noncoding introns.

### 7.6 Uro’gen decarboxylase

The conversion of uro’gen III into copro’gen III requires the decarboxylation of four acetic acid side chains at positions 2, 7, 12, and 18. These decarboxylations are catalyzed by a single enzyme named uro’gen decarboxylase (UroD). It is soluble, cytosolic, possesses no cofactor, and is a homodimer with subunit molecular weight of approximately 40,000 Da. UroD will catalyze the decarboxylation of both uro’gen I and III isomers. The enzyme has been isolated from a variety of organisms including human erythrocytes,^392,393^ bovine liver,^394^ avian erythrocytes,^395^ *R. sphaeroides*,^396^ yeast,^397^ *Chlorobium vibrioforme*,^398^ and *B. subtilis*.^399^ The gene for UROD was cloned from human spleen^400^ and its chromosomal location identified as chromosome 1p34.^400–402^

URO decarboxylase has been crystallized from human,^403,404^ tobacco,^405^ and bacteria.^399^ Its catalytic functions have been extensively studied by Phillips.^406–411^ The enzyme carries out the four decarboxylations in an ordered, sequential fashion starting with the D ring followed by the A, B, and finally C rings. The enzyme does this without any cofactor, yet Urod has been promoted as being a benchmark of proficiency with a calculated catalytic enhancement by a factor of over 10^17^.^412^

URO decarboxylase represents a significant evolutionary expansion of heme biosynthesis. The first “heme” synthesized by ancient organisms was siroheme, whose synthesis branches off after uro’gen III. That is also the same branchpoint for corrin synthesis. As detailed above and previously^3,206^ a limited number of organisms evolved the enzymatic machinery to convert siroheme into protoheme; the so-called AHB pathway. Two details of note are 1) the pathway through siroheme to protoheme is composed of oxygen-independent enzymes, and 2) no free porphyrinogens or porphyrins are synthesized in the AHB pathway. The evolution of UroD allowed for the creation of copro’gen that could be converted into protoheme in both CPD and PPD pathway containing organisms.

## 8 Enzymes of the CPD Pathway

As detailed above and previously^3,176^ the CPD pathway is found in Gram positive bacteria and is more ancient than the PPD pathway. Since its discovery, the CDP pathway has become the object of considerable research interest ranging from characterization of individual enzymes to delineation of regulatory mechanisms that appear unique to the CDP pathway (see ref 413).

### 8.1 Oxidation of Copro’gen III

The first step in the CPD is the oxidation of copro’gen into coproporphyrin. Two enzymes have been identified that catalyze this step. Aerobically this reaction is carried out by CgoX, an enzyme originally named HemY which at that time was believed to be a proto’gen oxidase.^184,185^ CgoX is highly similar to PgoX except that it is a soluble monomeric protein rather than a membrane associated homodimer. Both CgoX and PgoX possess a single noncovalently bound FAD in each monomeric unit. CgoX has been cloned and expressed (as HemY) from *B. subtilis*,^184,185^ *S. aureus*,^213^ *Mycobacterium tuberculosis* and *Cutibacterium* (formerly *Propionibacterium*) *acnes*.^176^ The enzyme from *B. subtilis* is best characterized^414,415^ and its crystal structure determined.^416,417^ The active site pocket of CgoX is twice the size of that of PgoX to accommodate the two additional propionate side chains present on copro’gen.^416^ The similarities and differences between CgoX vs PgoX are well reviewed by Zámocký et al.^418^

Anaerobically the oxidation of copro’gen to coproporphyrin III is catalyzed by the enzyme CgoN. CgoN was identified and characterized from *Priestia* (*Bacillus*) *megaterium*.^216^ The recombinantly produced CgoN is monomeric, possesses FAD as a cofactor and uses menadione *in vitro* as an acceptor for the six electrons. However, it has been shown in *S. aureus*, which lacks an identified gene for CgoN, that CgoX is able to support heme biosynthesis under nitrate-respiring, anaerobic culture conditions.^419^

### 8.2 Coproporphyrin Ferrochelatase

In the penultimate step of the CDP pathway ferrous iron is inserted into coproporphyrin III by the enzyme coproporphyrin ferrochelatase, CpfC. This enzyme was first identified and extensively characterized from *B. subtilis* as a protoporphyrin ferrochelatase by Hansson and colleagues before the CPD pathway was known.^185,420–423^ Unlike the PpfC enzymes, which are membrane associated dimers that do not utilize coproporphyrin III as a substrate, CpfCs are soluble monomers that will use both protoporphyrin IX and coproporphyrin III as substrates.^176^ The feature that accounts for this substrate specificity is a small loop that forms one lip of the active site mouth present in PpfC, but not CpfC. It has been shown in human ferrochelatase (FECH) that this lip encloses the active site during catalysis, thereby forming a snug pocket over the vinyl groups at the 2, 4 position of the A and B rings of the macrocycle of protoporphyrin IX.^424^ Some, but not all, CpfC possess [2Fe-2S] clusters.^425,426^ Unlike FECH, however, loss of this cluster in bacterial ferrochelatases does not necessarily result in the loss of catalytic ability.^425^

Since the discovery of the CPD pathway, CpfCs have received additional focus. CpfC from *Listeria monocytogenes* has been crystallized with and without appropriate substrates and products^427–429^ and a brief review of this enzyme is available.^430^ Additional studies for the *M. tuberculosis* ^431^ and *S. aureus* CpfC are published,^213,432^ but no crystal structures are currently available for these proteins.

### 8.3 Conversion of Coproheme into Protoheme

In the CPD pathway the terminal step is the oxidative decarboxylation of coproheme III into protoheme IX. The enzyme responsible for the catalysis under aerobic conditions is coproheme decarboxylase (ChdC).^175,176^ This water-soluble, homohexameric enzyme was originally annotated as a chlorite dismutase, an enzyme found in a limited number of prokaryotes (see refs 433 and 434 ). A role for the protein originally named HemQ in heme synthesis of Gram positive bacteria was first noted in 2010 ^209^ following a bioinformatics approach and the discovery that in *C. acnes* the gene for HemQ was part of a fusion in this organism with HemH. However, it was not until 2015 that its catalytic function was identified.^175,176,213^ ChdC may use both H_2_O_2_ and FMN as electron acceptors, leaving open the possibility that it may function in the absence of molecular oxygen as a terminal electron acceptor.^176^

Since its initial discovery as ChdC, considerable advances have been made, mainly by Hofbauer and colleagues (see ref 430). Recombinantly produced ChdC has been purified, crystallized and characterized from the firmicute *L. monocytogenes* ^435^ and the actinobacterium *C. diphtheria*.^436^ While it was initially noted that the amino acid sequences of HemQ were conserved within the Firmicutes and within the Actinobacteria, there exists significant sequence differences between these two groups.^175^ Structural and catalysis studies reveal that while both sets of enzymes catalyze the same reaction, differences exist in how the respective ChdCs interact with other pathway proteins.^436,437^

Under anaerobic conditions the conversion of coproheme III into protoheme IX may be catalyzed by the enzyme AhbD. This enzyme was first identified by Warren’s group as the terminal step in the AHB pathway to protoheme from siroheme^206^ and shares no similarity with ChdC. AhbD, also named heme synthase, has been characterized by Layer’s group as a radical SAM-containing enzyme that contains multiple [4Fe-4S] clusters and a SPASM domain.^438,439^ With the discovery of the CPD pathway, a bioinformatics analysis found that AhbD was found in some prokaryotes possessing the CPD pathway, most of whom lacked any other AHB pathway enzymes.^175^ So, while AhbD is an integral part of the more ancient siroheme-based AHB pathway, during evolution it was acquired by some Gram positive bacteria that contain the CPD pathway.

## 9 Enzymes of the PPD Pathway

From an evolutionary standpoint, the PPD pathway to heme is most recent. The largest change from the CPD pathway is that protoporphyrin IX, rather than coproporphyrin III, becomes the only porphyrin in the pathway. Additionally, protoheme, rather than siroheme or coproheme of the AHB and CPD pathways, respectively, is the direct product of metalation. One generally underappreciated aspect of the PPD is that it allowed for the existence of metal-free protoporphyrin IX, a compound essential for chlorophyll biosynthesis.^176^ However, as with so much in Nature, there appears to be an exception to this. The Gram positive heliobacteria^440^ lacks the PPD pathway yet synthesize a chlorophyll. While this conundrum remains to be settled, the proposal that early photosynthetic “chlorophylls” may have been Zn-containing protoporphyrin rather than the canonical Mg-containing tetrapyrrole,^441^ suggest the interesting proposition that CPD pathway enzymes may have been enlisted to create Zn protoporphyrin. This is a rather problematic since the terminal CPD pathway enzyme, ChdC, utilizes the coproheme iron to catalyze the decarboxylation of coproheme to protoheme.^442,443^ Alternatively, these bacteria may make protoheme via the CPD pathway and then remove the iron to yield protoporphyrin IX. This is clearly an area that deserves greater attention to resolve this question.

### 9.1 Oxidative Decarboxylation of Copro’gen III

In the first step of the PPD pathway, copro’gen III is converted into proto’gen IX by the oxidative decarboxylation of the propionate side chains into vinyl groups on the A and B pyrrole rings. The enzyme catalyzing this reaction is named copro’gen oxidase (**CpoX**) in eukaryotes. A homologous enzyme is found in some Gram negative bacteria (see above) and is named copro’gen decarboxylase (CpgD). CpoX is a homodimer with subunit molecular weights of approximately 35,000 Da in plants and yeast, and 40,000 Da in mammals. The enzyme possesses no cofactors.^444–446^ In most examined metazoans, the enzyme is located in the mitochondrion,^447,448^ but in yeast it is a soluble, cytosolic protein,^449^ and in plants it is located in the chloroplast.^450,451^ The plant and animal forms of the enzyme are synthesized in the cytosol with amino-terminal targeting sequences that are proteolytically removed upon translocation into the appropriate organelle. Based upon cell-free experiments with recombinantly produced CPOX, Susa et al.^452^ reported that 20 uM hemin inhibits the import of CPOX into isolated rat liver mitochondria. However, it was later shown that in intact cultured cells the targeting segment does not result in heme-controlled translocation under conditions where ALAS1 translocation into mitochondria is inhibited.^268^ The mature mammalian enzyme has an amino-terminal extension of approximately 100 residues that serves to tether the protein to the outside of the inner mitochondrial membrane.^453^ This segment is easily proteolytically nicked and has not been subject to investigation leaving open the question of whether CPOX needs to be tethered to the mitochondrial membrane to function in heme synthesis. The fact that yeast CpoX is soluble in the cytosol would suggest that it is not essential. However, if it is essential, then it would provide yet another site of regulation.

The reaction requires molecular oxygen and is sequential with the propionate of the A ring being decarboxylated before the B ring.^454–456^ The reaction goes via a monovinyl, monopropionyl deuteroporphyrinogen intermediate, referred to in most literature as harderoporphyrinogen, but this intermediate does not accumulate under normal conditions. Only the III isomer of copro’gen serves as a substrate. The enzyme mechanism was examined in considerable detail by the Lash and Jones research groups (see ref 456).

The gene for coproporphyrinogen oxidase has been cloned and expressed from human,^457^ mouse,^458^ yeast,^446^ soybean,^459^ barley and tobacco,^460^ *Plasmodium* ^461^ and several bacteria (see above). In human the chromosomal location is 3q12.^462^ The crystal structure of the yeast^446^ and human enzymes^444^ have provided clues as to the role played by catalytic residues.^463,464^

In anaerobic or facultative prokaryotic organisms, a separate enzyme has evolved to catalyze this reaction in the absence of oxygen. This “radical SAM” enzyme, named copro’gen dehydrogenase (CgdH), **is** detailed above. Crystal structures and catalytic studies on the recombinant enzyme have been published and are thoroughly reviewed.^16,217^

### 9.2 Oxidation of Proto’gen

The conversion of proto’gen IX into protoporphyrin IX is a six-electron oxidation of the noncolored, nonfluorescent, flexible cyclic tetrapyrrole into a fully conjugated, rigid planar molecule that is highly fluorescent and brightly colored. In eukaryotes this reaction is catalyzed by the FAD-containing enzyme proto’gen oxidase (PpoX)^465^ that in vitro consumes three molecules of molecular oxygen and produces three molecules of H_2_O_2_.^466^ In metazoans the enzyme is associated with the matrix side of the mitochondrial inner membrane.^467^ The enzyme has garnered considerable attention as an herbicide target in plants. Diphenylether types of compounds were initially studied as competitive inhibitors of PpoX^468–473^ causing free protoporphyrinogen to accumulate within plant cells. The porphyrinogen is oxidized in the plant cytosol to protoporphyrin which then acts as a photosensitizer in sunlight by generating singlet oxygen, thereby killing the cell. There are now many herbicides with similar properties (see ref 474). A considerable body of research has been published related to PpoX inhibitors. Indeed, there are over one hundred such publications.

The eukaryotic enzyme has been purified from mouse liver,^475,476^ and yeast.^477^ Early publications suggested that yeast PpoX is synthesized as a precursor with a molecular weight of 58,000 Da that is processed to 55,000 Da and subject to palymitoylation^478^ that was proposed to protect the protein from proteolytic degradation. Since that single publication, no other data have been presented to support this observation. However, it should be noted that all other structure-focused studies were done on recombinantly produced protein, which may lack this post-translational modification. The gene for the enzyme has been cloned, and the enzyme expressed and characterized from yeast,^479^ mouse liver,^480^ human placenta,^465,481,482^ and tobacco.^483,484^ In all cases the protein is dimeric, possessing one FAD per subunit, and having a subunit molecular weight of about 51,000 Da. The protein is expressed in the cytosol and translocated into the mitochondrion. Unlike many other mitochondrially located proteins, PPOX does not have a cleavable amino terminal leader sequence, nor does it have a heme binding domain.^268^ Instead, it has multiple internal segments required for efficient targeting. These include the region in the first 17 to 28 residues,^268,485,486^ as well an additional region located between residues 151 and 175.^487^ In humans the PPOX gene is localized to chromosome 1q22.23 ^488,489^ and in mouse it localizes to chromosome 1H2.^490^ Crystal structures have been determined for both tobacco^483^ and human^491^ enzymes.

As outlined above, in bacteria three different enzymes have been identified and characterized that carry out this reaction.^190^ PgoX, which is similar to eukaryotic PPOX, has been expressed and characterized from the bacterium *M. xanthus*,^186^ and has had its crystal structure determined.^492^ The gene for PgdH1 (HemG) has been cloned, and the protein expressed and characterized from *E. coli*.^181,182^ It resembles a flavodoxin with a single bound FMN, but there is no suggestion as to how it binds proto’gen or carries out its reaction. PgdH2 has been cloned and expressed,^187,188^ but little characterization of the protein has been done as of this date, and it has not been crystallized. What little is known is that it is a membrane associated, homodimeric *b*-type hemoprotein in *Synechocystis*.^189^

### 9.3 Protoporphyrin Ferrochelatase

The final step in the PPD pathway is insertion of ferrous iron into protoporphyrin IX. Ferrochelatase is a type II chelatase that requires only the two substrates in contrast with type I chelatases (such as Mg chelatase in chlorophyll synthesis) that require ATP for activity. *In vitro*, ferrochelatase will catalyze the insertion of Fe^2+^, Co^2+^, Zn^2+^, or Ni^2+^ into the dicarboxylate IX isomer porphyrins; proto-, hemato-, meso- and deuteroporphyrin. FECH is probably the best characterized pathway enzyme and has been purified from rat,^493^ bovine,^494^ chicken,^495^ mouse,^496^ and human.^497^ The gene has been cloned, and the protein expressed and characterized from *S. cerevisiae*,^498^ human,^499,500^ mouse,^501^ chicken and frog,^502^ *Schizosaccharomyces pombe*,^503^ cucumber,^504^ *Synechocystis*,^505^ and zebrafish.^506^ Genes encoding PpfC of the Gram negative bacteria *E. coli*,^507^ *Bradyrhizobium japonicum*,^508^ *Aquifex aeolicus*,^509^ *Caulobacter crescentus*,^431^ *M. xanthus*, *Pseudomonas putida*, and *P. aeruginosa* ^425^ have also been cloned, and the protein expressed and characterized. The recombinant enzyme has been crystallized from human^510^ and *S. cerevisiae*.^511^

In general the enzyme is a membrane-associated homodimer with a monomer molecular weight of approximately 42,000 Da. The metazoan enzyme is synthesized in the cytosol with an amino terminal, mitochondria-targeting sequence that is cleaved off after import into the mitochondrion^512^ where it is located on the inner surface of the inner mitochondrial membrane.^513^ This feature is lacking in all prokaryotic PpfC enzymes which are bound to the cytoplasmic membrane. In all metazoan FECH examined, and many prokaryotic PpfC,^426^ there is a carboxyl terminal extension that contains three of four cysteine residues that are involved in coordination of a [2Fe-2S] cluster.^514,515^ Some PpfC possess a [2Fe-2S] cluster that is coordinated by four internal cysteines.^425^ While the FECH cluster is sensitive to NO,^516^ an *in vivo* role of this cluster has not been defined. However, studies in zebrafish suggest that it may make the enzyme activity responsive to mitochondrial membrane potential.^506^ Additionally, since the metazoan FECH is only functional when the cluster is present, it has been proposed that iron availability may modulate FECH activity *in situ*.^517^

The best studied ferrochelatase is human FECH. The single gene for human FECH originally localized to chromosome 18q22 ^518^ and later to 18q21.3.^499^ Multiple crystal structures for wild-type and variants of FECH have been determined^424,510,519–523^ and from these studies a catalytic model has been proposed.^424,520,521,524^ In addition, evidence has been presented demonstrating that FECH is subject to posttranslational modifications that impacts enzyme activity.^525,526^

Plant ferrochelatases have been less well examined at a structural level, but a number of studies have demonstrated that plants produce two ferrochelatases. One, FC1, is found only in the mitochondrion of nongreening tissues, and a second, FC2, is found in plastids of all cells ( ^527,528^ for review). FC2 possesses a carboxyl-terminal extension that is similar to the chlorophyl a/b binding domain. This segment is essential for membrane binding and dimerization,^505^ and probably plays a role in regulation of heme synthesis.^505,528^

## 10 Spatial Organization of Pathway Enzymes

Critical to identifying all steps essential for heme biosynthesis was the ability to isolate and characterize individual enzymes. However, *in situ*, pathway enzymes do not float around in solution but exist in a gel-like matrix or associated with membranes, and, for some, in different cellular compartments. So, understanding the full functionality of the pathway requires that we understand the spatial distribution and potential protein–protein interactions of the pathway components.

Limited studies have examined spatial organization of prokaryotic heme synthesis. Enzymes of the CPD pathway are all soluble and localized in the cytoplasm, whereas in PPD pathway organisms, PgoX, PgdH2 and PpfC are membrane associated. As mentioned above, the formation of a protein–protein complex between the first two enzymes of the C-5 pathway have been characterized for *E. coli* ^302^ and *A. baumannii*.^295^ Additionally, evidence for an interaction between the two terminal PPD pathway enzymes, PpfC and PgoX, in *Thermosynechococcus elongatus* was demonstrated by immuno coprecipitation, and electron microscopy with immuno gold labeling.^529^ Similarly, a complex between PpfC and PgdH1 was reported for *Vibrio vulnificus*.^183^ In *Synechocystis* evidence was presented in support of an interaction between PgdH2 and CgoX.^189^ For the CPD pathway, evidence has been presented that supports an interaction between CpdC and CpfC (see ref 430) and in *C. acnes* one finds a fusion between CpfC and ChdC (HemH and HemQ).^209^ In *S. aureus* CpfC is found in a complex with IsdG, a heme oxygenase, which could possibly play a regulatory role for heme homeostasis.^530^ Additional protein–protein interactions between prokaryotic heme synthesis enzymes and proteins that serve a regulatory role are covered in excellent reviews by Beas et al.^531^ as well as Aftab and Donegan.^413^

In eukaryotes all heme synthesis pathway enzymes are nuclear encoded and synthesized in the cytosol. For metazoans, ALAS, CPOX, PPOX and FECH are post-translationally targeted to the mitochondrion where cofactor assembly occurs for ALAS, PPOX and FECH. As mentioned above, the localization of ALAS to the mitochondrion was noted by several groups and early work from Granick’s lab made preliminary assignment of some pathway enzymes to various cellular fractions in hemolyzed rabbit red cells.^117,118^ With the exception of protozoal parasites, metazoans examined to date have heme biosynthetic pathway enzymes organized with the first and (usually) last three enzymes in the mitochondrion and the intermediate four enzymes in the cytosol. The importance of proper cellular location for FecH in yeast was demonstrated by Prasad and Dailey^532^ who showed that FecH lacking its mitochondrial targeting sequence was localized to all cellular membrane fractions, but was not found in its usual location, the matrix side of the inner mitochondrial membrane. In these cells, the levels of mitochondrial cytochromes decreased by 40 to 60% in spite of total FecH levels being normal. Additionally, the stoichiometry of proteins can be of importance for proper pathway functioning. In yeast when FecH is overexpressed, one finds an increase in Zn-protoporphyrin production.^273^

The possibility that at least some of the heme synthesis pathway enzymes are spatially organized within the cell and may even physically interact with each other, was first broached for the enzyme copro’gen oxidase by Grandchamp et al. in 1978.^447^ They, along with Elder and Evans,^448^ located CPOX to the intermembrane space of the mitochondrion and suggested, without data, that CPOX not only catalyzed the conversion of copro’gen III to proto’gen IX, but may also transport proto’gen IX to the next pathway enzyme, PPOX, which Poulson and Polglase^134^ had localized to a mitochondrial membrane fraction. The first data-supported model for protein–protein interactions between heme synthesis enzymes came from work on the terminal two enzymes, PPOX and FECH.^533,534^ Kinetic data from mitochondrial membrane fractions vs solubilized enzymes was consistent with the presence of substrate channeling between these two enzymes. This work was expanded to examine the conversion of copro’gen to heme in isolated mouse mitochondria by Proulx, Woodard and Dailey.^535^ Their data demonstrated that while obligatory substrate channeling does not exist between these enzymes, under normal conditions they interact so as to ensure that the accumulation of pathway intermediates does not occur. Later, with the availability of crystal structures for FECH^510^ and PpoX^483^ it became clear that a stable, channeling complex between these terminal three pathway enzymes could not occur since each enzyme has a single active site “mouth” where substrate enters and product departs. Thus, interactions would, by necessity, be transient in nature.^275,524^ Additional participants in protein–protein interactions were identified by Furuyama and Sassa^536^ who demonstrated that ALAS2 interacted with the β-subunit of succinyl CoA synthetase (SUCLA2). This interaction occurs with ALAS2, but not ALAS1, and involves the carboxyl terminus of ALAS2.^289^ While one early suggestion was that this interaction was involved in supply of succinyl CoA for ALA synthesis, it is clear that this is not the case and that the interaction is likely involved in regulation of erythroid heme synthesis.^17,537,538^ Indeed, SUCLA2 was also found to interact with FECH.^273^

Amy Medlock’s group in the Department of Biochemistry and Molecular Biology at the University of Georgia^273^ carried out a number of targeted pull-down experiments that provided data in support of multienzyme protein complexes. While no evidence was obtained for complexes among the cytosolic enzymes, i.e., PBGS, HMBS, UROS and UROD, data supporting what was named a mitochondrial heme metabolon involving the mitochondrially located enzymes was garnered. Unexpectedly, reciprocal pulldowns revealed that ALAS and FECH have clear interactions, and this heme metabolon contains a variety of proteins that do not appear to have a direct involvement in heme synthesis^273,275,539^ (Figure 18). Current data support an interaction between the heme metabolon and mitochondrial membrane contact proteins.^540^ Abundant data now exist showing that FECH participates in a variety of protein pairings that involve iron metabolism, porphyrin synthesis, cellular metabolism, and mitochondrial membrane architecture.^273–275,524,539–541^ A number of these interactions and their role in cellular metabolism and regulation are reviewed by Yien and Perfetto.^18^ The recent identification of post-translational modification of some pathway enzymes,^17,525,526^ suggest that *in vivo* these modifications may also impact pathway functionality via protein–protein interactions.^17,542^

**Figure 18:**
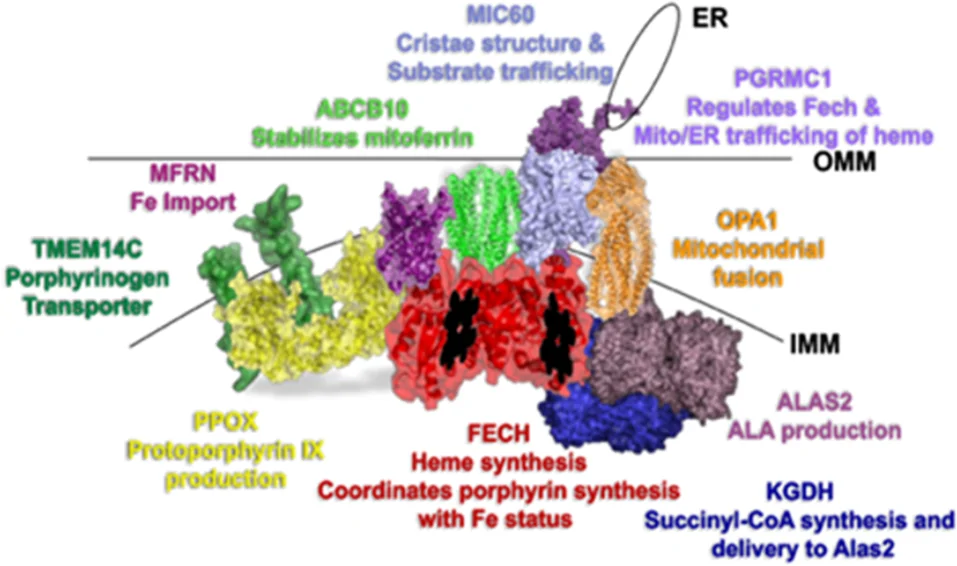
A model of the mitochondrial heme metabolon with key proteins and their roles in heme synthesis shown.

## 11 Unanswered Questions and Future Research Directions

### 11.1 Pathway Regulation

At this point in time, we now have a fairly complete knowledge of the individual steps that synthesize heme and the enzymes responsible for those steps. Among items currently lacking are descriptions and understanding of potential protein–protein interactions that may facilitate passage of intermediates along the pathway and even regulate the activity of other nonpathway enzymes such as α-ketoglutarate dehydrogenase.^538^ Additionally, the role of posttranslational modification (PTM) is something of a black box which is attributable in part to the use of non-native production of recombinant proteins for structure/function studies. The few studies that have been done suggest that protein–protein interactions (see refs 17 and 18 ) and PTM do play physiological roles in some organisms.^17,525^ Data from these fields of study will be essential to our understanding how heme synthesis is carried out and modulated *in situ*.

We have some knowledge of ways in which the pathway may be regulated for only a very limited number of organisms. However, it is clear that the canonical model where regulation of ALA synthesis alone is sufficient to modulate heme synthesis is no longer tenable for all cell types. At present our knowledge is limited and frequently confusing as over the years some have conflated data from multiple cell sources/growth conditions in an effort to create a uniform regulatory model. For prokaryotes it seems as if the number of regulatory mechanisms are as numerous and diverse as the number of species examined. Regulatory mechanisms will be distinct for photosynthetic vs nonphotosynthetic organisms, for organisms that synthesize cobalamin, or factor F_430,_ as well as for organisms adapted for nutrient rich vs nutrient poor environments. Even for the handful of prokaryotes examined, we do not have a comprehensive understanding of potential regulatory mechanisms.^3,543^ This will continue to be a field open for investigation as long as researchers continue to discover and characterize prokaryotes.

For multicellular eukaryotes, most attention on pathway regulation has focused on erythroid vs nonerythroid cell types in an assortment of mammals, and zebrafish. While there exists an impressive volume of research identifying numerous participants in pathway regulation in these systems, we still lack a comprehensive regulatory model. Indeed, every year sees more players proposed. The two best studied features, the modulation of ALAS1/2 and the supply of iron at the cellular level, still lack critical data. Additionally, the observation from Kushner and Burnam’s studies discussed above on a possible role for DOVA via AGXT2 in the biosynthesis of ALA has long been forgotten, but perhaps worth reinvestigating as a noncanonical pathway to ALA in some cell types. Total body and cellular iron metabolism are relatively well studied, but it is not clear for eukaryotes exactly how appropriate iron supply to ferrochelatase for heme synthesis is modulated or accomplished. For many of the currently identified proposed factors, it is not obvious which are specific for the heme biosynthetic pathway and which have more global effects at a cellular level. Studies in typical cell culture, or tissue homogenates lack essential structural architecture, cell–cell interactions, and circadian fluctuations found in whole organs in animals. Temporal and spatial features are critical but poorly characterized at present. There is much to be learned in the coming years.

Heme synthesis is not universal among all hemoprotein-containing organisms,^1^ and it is becoming increasingly clear that within some, perhaps all, multicellular organisms, heme transport between cells may be essential for whole body heme homeostasis.^20^ To date, most research has focused on a limited number of experimental cell types grown as homogeneous cultures in synthetic media that bear little resemblance to the natural environment. This is clearly an area that will receive increasing attention in the coming years and is bound to upend some long-held beliefs about the localization of heme biosynthesis.

### 11.2 Coproporphyrin Production

One outstanding question related to heme biosynthesis across all organisms is why do cells produce, accumulate, and excrete coproporphyrin I or III. It would seem that for such an ancient biosynthetic pathway, evolution would have minimized the cost of synthesis and not have wasteful overproduction of coproporphyrin. Yet one finds copro “overproduction” as the rule rather than the exception. Even in mammals where pathological accumulation of copro I can result in the disease coproporphyria, coproporphyrin production and excretion is most frequently considered normal.^544^ It would seem logical to posit that coproporphyrin plays some significant yet undefined role at the organismal level. But at present there are no data to indicate what this role may be. Bacteria are also known to produce, accumulate and excrete coproporphyrin, even to the level where it may cause blue light-induced cell death via photodynamic-mediated cell destruction. This may occur via naturally synthesized coproporphyrin,^545–547^ or in cells where coproporphyrin levels have been increased by intervention with exogenously supplied ALA or a small molecule that stimulates coproporphyrin production.^548^ Interestingly, Rebecca Donegan’s group in the Department of Chemistry, Barnard College, Columbia University, recently reported that in *Mycobacterium*, which possesses the CPD pathway, there is no direct link between iron availability, or heme production, and coproporphyrin accumulation/excretion.^549^ This supports a model where free coproporphyrin is produced and regulated for a specific purpose and not just as the result of an inadequately regulated heme synthesis pathway. Assuming that coproporphyrin is not being produced as a suicide molecule, it must serve some useful role. It has been proposed that coproporphyrin may be serving a function in copper^550^ or zinc^551^ acquisition. In natural environments it may also function in symbiotic relations with other organisms. This was shown to be the case where coproporphyrin produced by *C. acnes* induces biofilm formation by *S. aureus*.^552^ Or it may be that the excreted porphyrin acts as an antimicrobial agent targeting microorganisms that have the CDP pathway. Assuming that CpfC can metalate coproporphyrin I and that the metalated coproheme I is inappropriately metabolized by ChdC, coproporphyrin I may serve as a Gram positive targeted antimicrobial. More work is required before there are answers to this question.

### 11.3 Substrate Availability and Byproduct Fate

One issue seldom considered is the source of substrates for ALAS and the impact that this may have on cellular metabolism during periods of increased heme synthesis. It is generally assumed that succinyl CoA is provided by the TCA cycle although there are no published data to directly support this proposal. While it is conceivable that cells synthesizing relatively small amounts of heme may draw succinyl CoA from the TCA cycle, for developing erythroid cells which produce approximately 10^9^ molecules of heme per cell in a short period of time, this would create a great strain on the cell. Burch et al.^537^ demonstrated that in these cells the succinyl CoA for heme synthesis is provided from glutamine via α-ketoglutarate dehydrogenase (KDH) operating outside of the TCA cycle. Additionally, it has been found that KDH exists in a mitochondrial heme metabolon with ALAS and FECH.^273,537^ Recently Raven and associates have examined heme homeostasis and its significant impact on global cellular metabolism.^8^ However, to date, no studies have been published to demonstrate the fate of the carboxyl groups released during protoporphyrin biosynthesis. While this may seem a trivial issue, it should be remembered that during the synthesis of a single molecule of heme, eight molecules of CO_2_ are released in the mitochondrial matrix during the formation of the required eight ALA molecules. Four additional CO_2_ are released during the decarboxylation of uro’gen to copro’gen in the cytosol, and another two from the oxidative decarboxylation of two propionates to vinyl groups in forming proto’gen from copro’gen in the inter mitochondrial membrane space. Thus, a total of 14 CO_2_ (representing 29% of the total carbon atom input) are released along with four glycine-derived NH_4_, six electrons from the oxidation of proto’gen and two additional electrons from insertion of iron during the synthesis of one molecule of heme via the ALAS (C4) based pathway. The cellular fate of the carbon and nitrogen atoms is currently unknown. Presumably they are recycled into other cellular components, but the possibility that they serve a signal/regulatory function has never been explored.

### 11.4 Heme as an Intermediate

It is frequently forgotten that heme, in addition to being the end product of the pathway, is also an intermediate in other pathways. Most organisms possess the ability to oxidatively break the cyclic tetrapyrrole into a linear tetrapyrrole. Among many prokaryotes and eukaryotes this is an essential mechanism to obtain iron from dietary heme. Among all eukaryotes this degradation results in the production of biliverdin and the release of iron and carbon monoxide. Mammals take this one step further and convert the water-soluble biliverdin into the lipid-soluble bilirubin via the action of the enzyme biliverdin reductase. Some photosynthetic organisms produce and utilize linear tetrapyrroles, bilins, as accessory light-harvesting pigments which are bound to phycobiliproteins.^553,554^ These compounds arise from the oxidative cleavage of heme so requirements to regulate the pathway must exist to produce appropriate amounts of heme to serve in hemoproteins as well as a precursor to phycobiliproteins. Given the value of these compounds as food additives and potentially in therapeutic applications, examination of the cellular regulatory mechanisms existing for their production is an area of active research. Tetrapyrroles, most frequently protoporphyrin and/or biliverdin, also are found in numerous avian egg shells and as colorants in some mollusks. The current understanding of how the synthesis of these compounds is regulated is a black box.

## 12 Summary

The story of heme biosynthesis research is a chronicle of biochemical discovery, as well as a testament to technological advances. From the earliest chemical isolations of porphyrins to the modern integration of genomics, structural biology, and evolutionary perspectives, each succeeding generation of researchers has refined their experimental approaches with an ever-increasing variety of tools. This history illustrates how scientific progress is rarely linear. Indeed, ALA was identified as the initial compound in the pathway only after the structure of PBG was determined, and the characterization of how the III isomer of uro’gen was formed came decades after all of the enzymes of the classical pathway were identified. Today, the field advances via structural biology, systems biochemistry, and evolutionary genomics, with new questions emerging about pathway regulation, metabolon organization, and intercellular and intracellular heme trafficking. What began as a pursuit to understand the red pigment of blood has grown into a multidisciplinary field that illuminates fundamental principles of metabolism, disease, and adaptation. By revisiting the intellectual journey that brought us here, we acknowledge the ingenuity and hard work of past investigators, and gain perspective on how future discoveries may further shape our understanding of this ancient and indispensable molecule.

## Abreviations

ALA: 5-aminolevulinate
ALAS: 5-aminolevulinate synthase
PBG: porphobilinogen
PBGS: PBG synthase
HMB: hydroxymethylbilane
HMBS: HMB synthase
URO: uroporphyrin
UROS: uro’gen, uroporphyrinogen
UROS: uro’gen synthase
UROD: uro’gen decarboxylase
COPRO: coproporphyrin
copro’gen: coproporphyrinogen, CPOX, copro’gen oxidase, PROTO, protoporphyrin IX
proto’gen: protoporphyrinogen
PPOX: proto’gen oxidase
FECH: ferrochelatase
SAM: S-adenosylmethionine
